# Blood and cerebrospinal fluid flow oscillations measured with real-time phase-contrast MRI: breathing mode matters

**DOI:** 10.1186/s12987-022-00394-0

**Published:** 2022-12-14

**Authors:** Maria Marcella Laganà, Sonia Di Tella, Francesca Ferrari, Laura Pelizzari, Marta Cazzoli, Noam Alperin, Ning Jin, Domenico Zacà, Giuseppe Baselli, Francesca Baglio

**Affiliations:** 1 IRCCS Fondazione Don Carlo Gnocchi ONLUS, Milan, Italy; 2 Department of Psychology, Università Cattolica del Sacro Cuore, Milan, Italy; 3 University of Miami, Miami, USA; 4 MR R&D Collaborations, Siemens Medical Solutions USA, Inc, Cleveland, OH USA; 5 Siemens Healthcare, Milan, Italy; 6 Department of Electronics, Information, and Bioengineering, Politecnico di Milano, Milan, Italy

**Keywords:** MRI, Phase-contrast, Real-time, Blood flow, Cerebrospinal fluid flow, Respiration

## Abstract

**Background:**

Cervical blood and cerebrospinal fluid (CSF) flow rates can be quantified with Phase-contrast (PC) MRI, which is routinely used for clinical studies. Previous MRI studies showed that venous and CSF flow alterations are linked to various pathological conditions. Since it is well known that, besides the heart beating, the thoracic pump influences the blood and CSF dynamics, we studied the effect of different respiration modes on blood and CSF flow rates using a real-time (RT)-PC prototype.

**Methods:**

Thirty healthy volunteers were examined with a 3 T scanner. A RT-PC sequence was acquired at the first cervical level to quantify the flow rates of internal carotid arteries, internal jugular veins (IJVs) and CSF. Each RT-PC acquisition was repeated three times, while the subjects were asked to breathe in three different ways for 60 s each: freely (F), with a constant rate (PN) and with deep and constant respiration rate (PD). The average flow rates were computed, they were removed from the respective signals and integrated in the inspiratory and expiratory phases (differential volumes). Finally, the power spectral density was computed for each detrended flow rate. High- and very-high frequency peaks were identified on the spectra while their frequencies were compared to the respiratory and cardiac frequencies estimated using a thoracic belt and a pulse oximeter. The area under the spectra was computed in four 0.5 Hz-wide ranges, centered on the high-frequency peak, on very-high frequency peak and its 2nd and 3rd harmonics, and then they were normalized by the flow rate variance. The effect of breathing patterns on average flow rates, on systolic and diastolic peaks, and on the normalized power was tested. Finally, the differential volumes of inspiration were compared to those of expiration.

**Results:**

The frequencies of the high- and very-high spectral peaks corresponded to the respiratory and cardiac frequencies. The average flow rate progressively decreased from F to PN to PD breathing, and the cardiac modulations were less predominant especially for the IJVs. The respiratory modulation increased with PD breathing. The average volumes displaced in the inspiratory phases were not significantly different from those of the expiratory one.

**Conclusions:**

The spectral analyses demonstrated higher respiratory modulations in PD compared to free breathing, even prevailing the cardiac modulation in the IJVs, showing an increment of the thoracic pump affecting the flow rate shape.

**Supplementary Information:**

The online version contains supplementary material available at 10.1186/s12987-022-00394-0.

## Background

Brain functioning depends on cerebral circulation, which supplies oxygen and nutrients to the cerebral tissues and removes waste [1]. Therefore, an effective arterial flow to the brain and venous outflow are fundamental for a healthy brain. Brain waste clearance also depends on the cerebrospinal fluid (CSF) flow, connected to the recently described glymphatic system [2]. Arterial, venous and CSF flow cyclic oscillations depend on many factors, such as heartbeat, posture [3, 4], physical effort [5], and respiration. Cardiac-related modulations have been described for decades [6–8]. Recent studies suggested that the thoracic pressure changes during the different respiratory phases influence the CSF [9, 10], arterial [11], and venous flows [4, 12]. Understanding the physiologic role of respiration for the blood and CSF dynamics is essential for testing the potential effect of respiratory pathologies on brain health. For example, recent studies showed that chronic obstructive pulmonary disease patients with diverse lung impairment exhibited progressive brain hypoperfusion [13] and structural damage [14], with consequences on cognitive performances [13].

The arterial and venous flow in/to the brain and the CSF flows can be quantified with Phase-contrast (PC) MRI [15–17]. PC-MRI is routinely utilized in normal pressure hydrocephalus for diagnosis and for the prediction of the response to surgery [18]. Its use in clinical studies revealed venous and CSF alterations linked to various pathological conditions [19–21]. However, standard cine PC-MRI is based on cardiac gating, and it requires several heartbeats to form the time-resolved flow images covering the entire cardiac cycle. Therefore, with cardiac-gated PC-MRI, it is not possible to detect the beat-by-beat changes in flow rates and the influence of respiration.

The recent advent of PC-MRI techniques for quantifying flow in real-time (RT) overcame this limitation and showed that coughing, inspiration and expiration all have an effect on venous [12, 22], and CSF [12, 22–25] flows. Specifically, a study by Dreha-Kulaczewski and colleagues [26] showed that forced inspiration increases the venous return that is counterbalanced by a cranial CSF flow. Conversely, during forced expiration, the CSF flow quantified at a high cervical level is very low. As a consequence, the net CSF flow volumes were directed upward during forced inspiration (cranial net flow during inspiration, plus low volume during expiration). Consistently, another work from the same group [12] showed that the average flow rate (mean value across all the time frames) was higher during forced compared to normal respiration. Moreover, Ohno et al. [22] showed that the CSF pressure gradient was higher during full inspiration breath holding than at the end of expiration and during free breathing. Finally, the effects of forced breathing compared to normal respiration [12] revealed venous flow decrement and the respiratory modulation increment. However, to the best of our knowledge, none of the published studies assessed the effect of respiration on arterial flow, besides venous and CSF flows, using RT-PC MRI.

In our study, we examined the cervical arterial, venous and CSF flow rates using RT-PC MRI with the following aims: (1) testing the presence of the cardiac and respiratory drivers; (2) assessing how different modes of breathing (i.e. free, paced, paced and forced) affect the (A) mean, systolic and diastolic peaks of the flow rate, and the (B) respiratory and cardiac modulations to the flow rate; (3) testing whether the flow volumes are different between inspiration and expiration in different breathing modes.

We previously described our protocol in a preliminary study [27], where we showed that the forced paced breathing, compared to a normal paced breathing, increased the respiratory modulations to arterial, venous and CSF flow rates. In the current study, we increased the sample size to 30 healthy volunteers and we completed the protocol acquiring the subjects during a rest condition, i.e., while they breathed freely, as is routinely done for cardiac-gated PC-MRI.

## Methods

### Subjects

We recruited 30 healthy volunteers, with no contraindication to the MRI exam, no history of cardiovascular diseases, brain tumours, diabetes, neurologic or psychiatric diseases.

### MRI acquisitions

All the subjects were scanned with a 3 T MR scanner (MAGNETOM Prisma, Siemens Healthcare, Erlangen, Germany) equipped with a 64-channel head-neck coil.

The flows were estimated using a prototype RT-PC [24] with a segmented EPI readout, parallel acceleration factor in the temporal direction, and 2-sided shared velocity encoding reconstruction algorithm. Each RT-PC acquisition lasted 60 s, and had a FOV of 153 × 175mm^2^, a matrix of 96 × 128, interpolated to an in-plane resolution of 0.7 × 0.7mm^2^, with a slice thickness of 8.6 mm. For the neck blood flow measurement, the imaging slice was placed perpendicular to the main neck vessels, and the following parameters were set: temporal resolution = 58.5 ms, velocity encoding (VENC) = 70 cm/s, GRAPPA = 3, TR/TE = 14.6/8 ms, flip angle = 15°. For the CSF flow quantification, the imaging slice was placed orthogonal to spinal cord, with temporal resolution = 94 ms, VENC = 6 cm/s, GRAPPA = 2, TR/TE = 15.7/9 ms, flip angle = 5°.

**Fig. 1:**
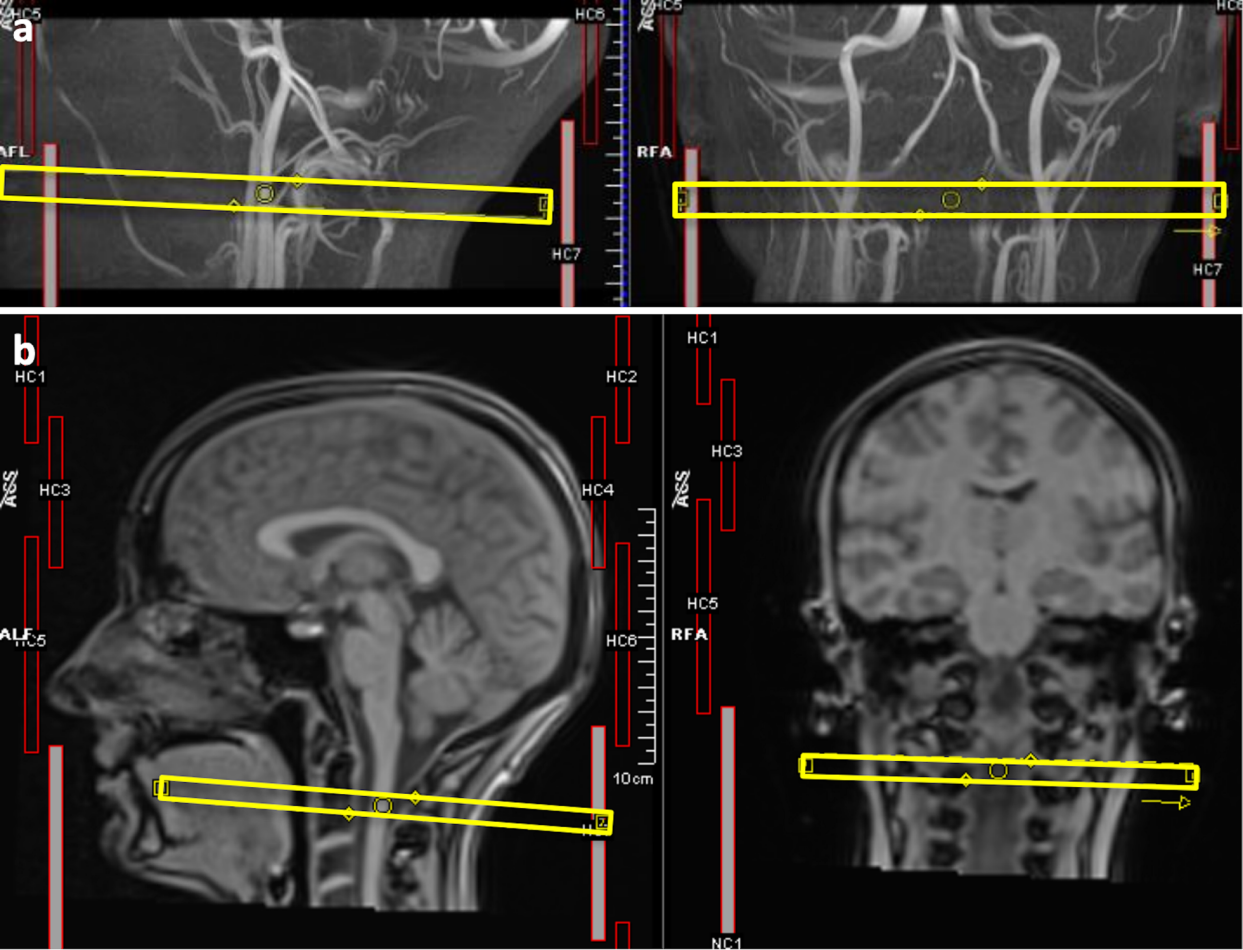
Slice positioning. **a** slice for the blood (VENC = 70 cm/s) acquisition positioned perpendicularly to neck vessels seen in the sagittal and coronal Maximum Intensity Projection of a time-of-flight sequence; **b** Cervical levels seen on sagittal and coronal localizers, for positioning the RT-PC sequences

The blood and CSF flows were measured at the first cervical level. The sagittal and coronal Maximum Intensity Projection of a time-of-flight sequence (169 axial slices, FOV = 200 × 180mm^2^; axial resolution = 0.3 × 0.3 mm^2^; slice thickness = 0.5 mm; TR = 21 ms; TE = 3.42 ms; flip angle = 18°) at the neck level allowed to position the slice for the cervical blood flow measure, perpendicularly to Internal Jugular Veins (IJVs) and Internal Carotid Arteries (ICAs) as in [28] (Fig. 1a). To identify the cervical level for positioning the RT-PC acquisition plane, and to position the slice for the CSF acquisition perpendicularly to the spinal canal, sagittal and coronal localizers were used (Fig. 1b).

Each RT-PC was acquired three times, in the following conditions: (1) free breathing (F), i.e., asking the subject to breathe at his/her natural rate; (2) paced breathing (PN), i.e., the subject was instructed to breathe at a constant rate as explained hereafter; (3) paced and forced (deep, PD) breathing, i.e., as the previous condition, but with forced inspirations and expirations. To maintain a constant breathing rate (BR) in conditions (2) and (3), we used a visual stimulus consisting of a circle with enlarging/decreasing diameter as a guidance. Both the BR of (2) and (3) were adapted to the subject. Regarding (2), we imposed the spontaneous mean BR measured in (1). In condition (3), we asked the subject to perform a few forced breaths and then adapted the stimulus to this new BR to allow the subjects to self-compensate by lower BR the higher tidal volume. Breathing and heart pulse (physiological signals) were recorded using a belt and a pulse oximeter during the RT-PC acquisition.

### Scheme of the whole processing

**Fig. 2:**
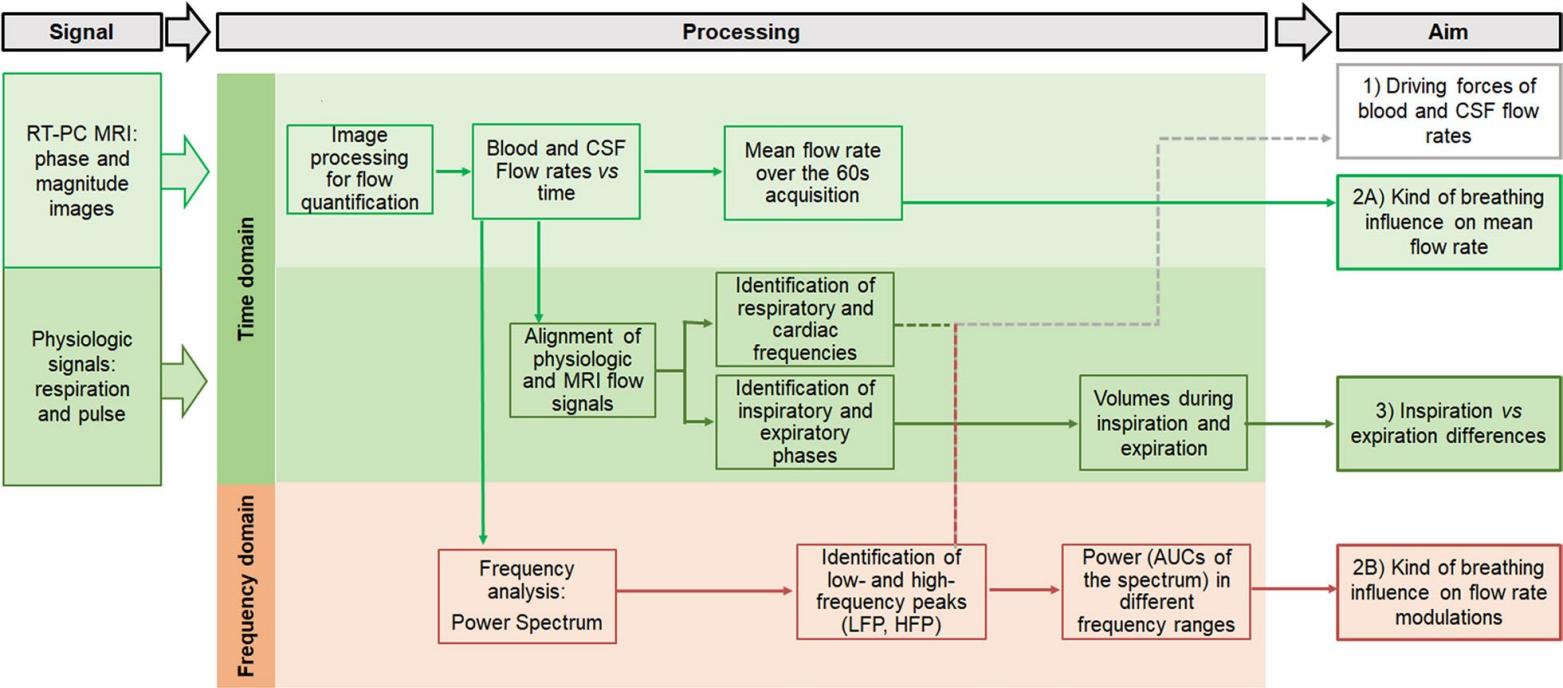
Scheme of the Real-Time Phase-Contrast (RT-PC) MRI data and physiological signals processing

The RT-PC MRI data and the physiological signals were processed as schematized in Fig. 2, for addressing the three aims of this work.

All steps will be detailed in the following paragraphs (MRI Data Processing, Signal analysis, Statistical analysis) and are summarized hereafter. The physiologic signals were aligned to the MRI data, to estimate the BR and HR during each acquisition and to identify the respiratory phases. Then, the RT-PC MRI data were processed for quantifying cervical arterial, venous and CSF flow rates. The analysis in the time domain consisted of computing the average flow rates (in ml/s) over time and the volumes (in ml) over the inspiratory and expiratory phases. The former was compared among the different breathing modes to address aim 2A. The latter was compared between respiratory phases to address aim 3. The analysis in the frequency domain consisted of computing the power spectrum, identifying its various peaks, and estimating the power in different frequency ranges. The comparison of the peak frequencies and the respiratory and cardiac frequencies estimated from the physiologic signals allowed to address aim 1. The power in different frequency ranges was used to test if there were differences of the respiratory and cardiac modulations due to the different breathing type (aim 2B). Conventional [29] nomenclature was used for defining the frequency ranges we focused on: high (0.15–0.6 Hz) and very high (0.6–2 Hz) frequencies (HF and VHF respectively).

### MRI data processing

RT-PC scans were processed using signal processing in NMR (SPIN) software (SpinTech Inc, Bingham Farms, MI) [30] by a single trained operator. After the time frame with the highest flow was visually selected, the phase and magnitude images were magnified so that the vessels of interest or the CSF were well visible on the screen. Regions of interest (ROIs) corresponding to the ICAs, IJVs, and CSF were drawn using a semiautomated method [30], consisting of the manual identification of a pixel inside the structure of interest and then in the application of region growing. Four regions of static tissue (no-flow areas—NFA) were manually drawn near the ROIs and used to estimate the background phase within the ROIs for background phase correction. ROIs were copied to all the time frames and manually adjusted if needed. The phase values of each ROI were corrected for the phase offset derived in the NFA, and then the average velocity was computed inside each ROI. By convention, velocities directed upward, i.e., positive in the cranial direction. Each ROI area was computed and multiplied by the average velocity inside the ROI itself to estimate the average flow rate in ml/s of each time point. The flow rate of the left and right ICAs, and left and right IJVs, were separately summed. The average ICAs, IJVs, and CSF flow rates [31], the CSF absolute flow rate, were obtained for each subject and each type of breathing averaging the flow rates over all the time points in the 60-s acquisition. The mean systolic and diastolic peaks were computed identifying all the peaks and averaging them over each acquisition.

The average processing time was 10 min for each subject.

### Signal analysis

The following processing was performed using ad-hoc scripts in MATLAB (version 2021a, Mathworks, Natick, WA, USA).

Using the image acquisition time stored in each DICOM header, and the start time stored in the log files recorded from the pulse oximeter and thoracic belt, we could temporally align the physiologic signals to the MRI data. The average BR and HR during each acquisition were then computed, and the respiratory phases were identified in the respiratory signal.

After 4th grade polynomial signal detrend of each flow rate, the power spectral density was computed, separately for ICAs, IJVs and CSF flow rates, in the three breathing conditions. For the blood, the signal detrend avoided the presence of a high peak around the zero-frequency, due to the average flow rate. Even if CSF oscillates around zero, for consistency we performed the same signal analysis as for the blood flow rate. For each spectrum, two fundamental peaks were recognized: a HF peak around 0.2 Hz, and a VHF peak around 1 Hz. Other three peaks were identified, corresponding to the second HF peak harmonic and to the second and third VHF peak harmonics. We then defined a HF band as the interval from 0.15 Hz before the HF peak to 0.35 Hz after the HF peak, and three cardiac bands, centred in the VHF peak [12, 24], its 2nd and 3rd harmonic respectively, with a width of 0.50 Hz as in the respiratory one. The HF band included also the second HF peak harmonic. The power in each range of frequencies was computed as the area under the curve (AUC) of each band. Then, the following indices were derived: (i) the classic relative contribution of the main respiration versus cardiac component (AUC of the respiratory band divided by the AUC of the first cardiac band) [12, 24]; (ii) the AUC of the respiratory band, and (iii) the AUC of the three cardiac bands, both normalized by the whole signal variance, obtaining normalized powers (values from 0 to 1).

**Fig. 3:**
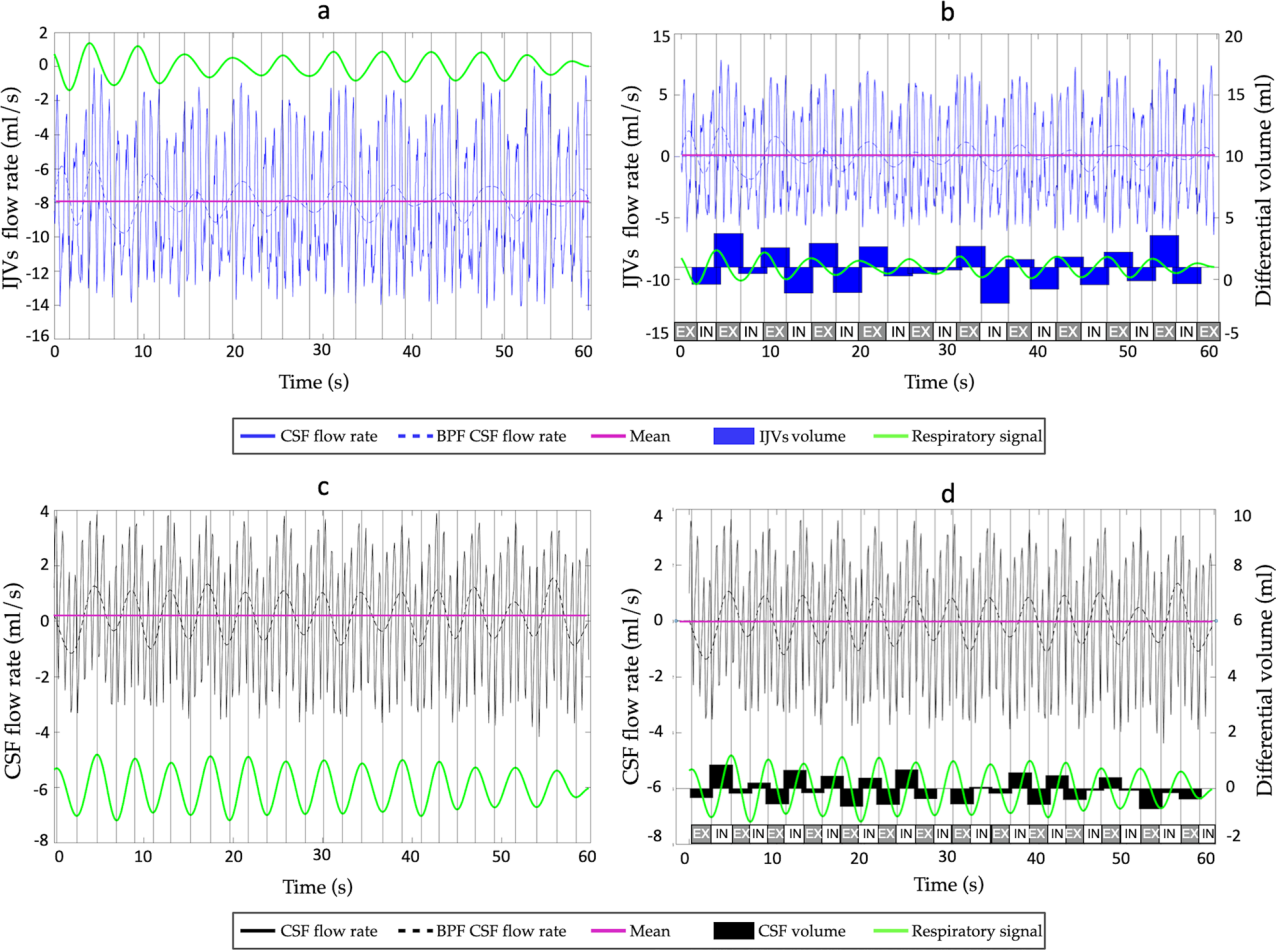
Differential flow volume (in ml) computed during each inspiration and expiration. Respiratory signal (green), flow rate curve and its mean value (purple) are shown in (**a**) and (**c**) for the Internal Jugular Veins (IJVs—blue curve) and cerebrospinal fluid (CSF—black curve) respectively. In **b** and **d**, the mean-centered flow rates are shown (flow rate curves minus its mean value) for the IJVs (**b**) and CSF (**d**). The inspiration (in) and expiration (ex) phases are written, based on the respiratory signal: its rising parts were considered inspirations. The differential volumes (integrals of the mean-centered flow rate over the inspiratory and expiratory phases) are shown as black bars in (**b**) for the IJVs and (**d**) for the CSF

The differential flow volume (in ml) during each inspiration and expiration was computed as follows. First, we removed the mean from the flow rate curve (mean-centered flow rate, Fig. 3 from a to b) Then, we selected the phases corresponding to inspiration and expiration (Fig. 3, in, ex respectively), based on the respiratory signal acquired with the thoracic belt: the rising parts of the signal, i.e., from minimum to maximum, were considered inspirations (thorax expansion); and the others were expirations. Finally, we computed the integrals of the mean-centered flow rate over the inspiratory and expiratory phases (Fig. 3b). They can be interpreted as follows: a positive value means more flow volume compared to the mean in the cranial direction, negative value means more flow volume in the caudal direction. The average inspiratory and expiratory mean-centered flow volume (in ml) was finally computed.

### Statistical analysis

The statistical tests were performed using Jamovi version 2.2 (The jamovi project (2021), https://www.jamovi.org).

The normality of data distribution was checked using the Kolmogorov–Smirnov test. Then, the variables that were not normally distributed were transformed using the natural logarithm transformation. Finally, normality was re-assessed after transformation to ensure that they were subsequently normally distributed. Descriptive statistics were expressed as means and standard deviations for the normally distributed variables; median [range] were used otherwise.

In order to assess if the breathing mode influenced the respiratory and cardiac frequencies, the frequencies were compared among breathing modes using Repeated-Measures Analysis of Variance tests (RM-ANOVA).

For aim 1, we conducted the following tests. Three-way RM-ANOVAs were used to test if the spectral HF peak and its 2nd harmonic were statistically different compared to the respiratory frequency (estimated with thoracic belt) and double its value, and if there was an effect of the breathing mode. The RM-ANOVA was conducted separately for the spectral peaks estimated from ICAs, IJVs, and CSF flow rates. For each RM-ANOVA, we tested the interaction between estimation method (i.e., frequency evaluated using the thoracic belt or frequency estimated from the spectra), peak number (i.e., first or second harmonic), and respiration type (i.e., F, PN, or PD). As for HF peak, three-way RM-ANOVAs were used to test if the spectral VHF peak and its 2nd and 3rd harmonic were not statistically different compared to the cardiac frequency, estimated with the pulse oximeter, and double as well as triple its value, and if there was an effect of the breathing mode.

For aim 2, two-way RM-ANOVAs were used to test if the kind of respiration and the kind of structure of interest had an effect on the average flow rates, systolic and diastolic peaks, ROI areas, on the cardiac and respiratory components and on their ratio (R/C). In particular, we used average flow rates, areas, cardiac and respiratory components and R/C as dependent variables separately in each RM-ANOVA, and we tested the interaction between the respiration mode (i.e., F, PN, PD) and the structure of interest (i.e., ICAs, IJVs, CSF). For the detailed analysis of the cardiac component, i.e., the separate study of its three peaks, we also conducted a three-way RM-ANOVA to test if the kind of respiration, the kind of structure of interest, and the peak frequency range had an effect on the normalised AUC. In particular, we used normalized AUCs as dependent variables and we tested the interaction between the respiration type (i.e., F, PN, PD), the structure of interest (i.e., ICAs, IJVs, CSF), and the peak range (i.e. [− 0.15 Hz – + 0.35 Hz] for HF peak, 0.5 Hz-wide windows centered in the VHF peak and its 2nd and 3rd harmonics).

For aim 3, we used three-way RM-ANOVAs in order to test if the kind of respiration, the kind of structure of interest, and the respiratory phase had an effect on the fluid volumes. In particular, we used fluid volumes as dependent variables and we tested the interaction between the respiration type (i.e., F, PN, PD), the structure of interest (i.e., IJVs, CSF), and the respiratory phase (i.e., inspiration and expiration).

All the post-hoc comparisons were adjusted for multiple comparisons using the Bonferroni correction. Corrected p-values lower than 0.05 were considered significant.

## Results

### Subjects

Thirty healthy subjects (21 females, median age = 26 years old, age range = 19–57 years old) were included the current study.

The respiratory frequency decreased from F to PN to PD breathing (p < 0.01 for all the comparisons), while the cardiac frequency did not change with the type of breathing (Additional file 7: Table S1).

### Respiratory, cardiac frequencies and spectral peaks (aim 1)

No significant difference was observed between the main HF peak obtained from the spectra and the BR measured with the thoracic belt (Additional file 7: Table S2). The 2nd HF peak harmonic of ICA and of CSF spectra in F breathing was significantly different from 2*BR (p < 0.001). All the other comparisons between HF peak harmonics obtained from the spectra and the multiple of BR measured with the thoracic belt did not show any significant difference.

No significant difference was observed between the main VHF peak obtained from the spectra and the HR measured with the pulse oximeter (Additional file 7: Table S3). The 3rd VHF peak harmonic of ICA spectrum in PN breathing was significantly different from the 3* HR measured with the pulse oximeter (p = 0.009). All the other comparison between VHF peak harmonics obtained from the spectra and the multiple of HR measured with the pulse oximeter did not show any significant difference.

Mean flow rate changes with type of breathing (aim 2A).

**Fig. 4:**
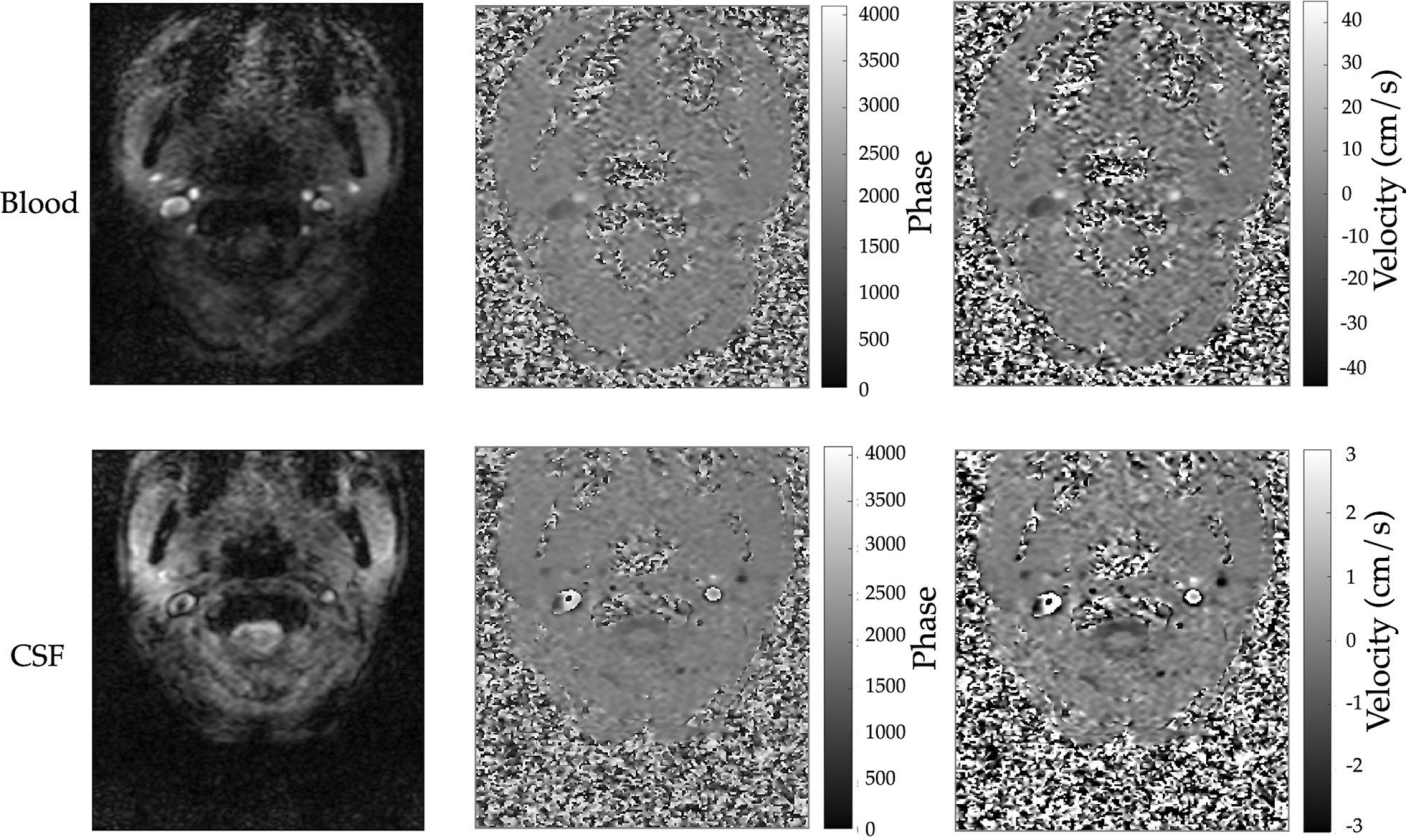
Data obtained with the real-time (RT) Phase Contrast (PC) sequence. Exemplificative magnitude (**a**, **d**) and phase (**b**, **e**) images obtained from the RT-PC with velocity encoding (VENC) of 70 cm/s (**a**, **b**) for the blood flow quantification, and VENC = 6 cm/s (**d**, **e**) for the cerebrospinal fluid (CSF) flow quantification. Velocity map of the blood (**c**) and CSF (**f**) acquisitions

Exemplificative images used for estimating the blood and CSF flows are reported in Fig. 4, where magnitude (Fig. 4a and d), phase (Fig. 4b and e), and velocity maps (Fig. 4c, Fig. 4f) are reported.

**Fig. 5:**
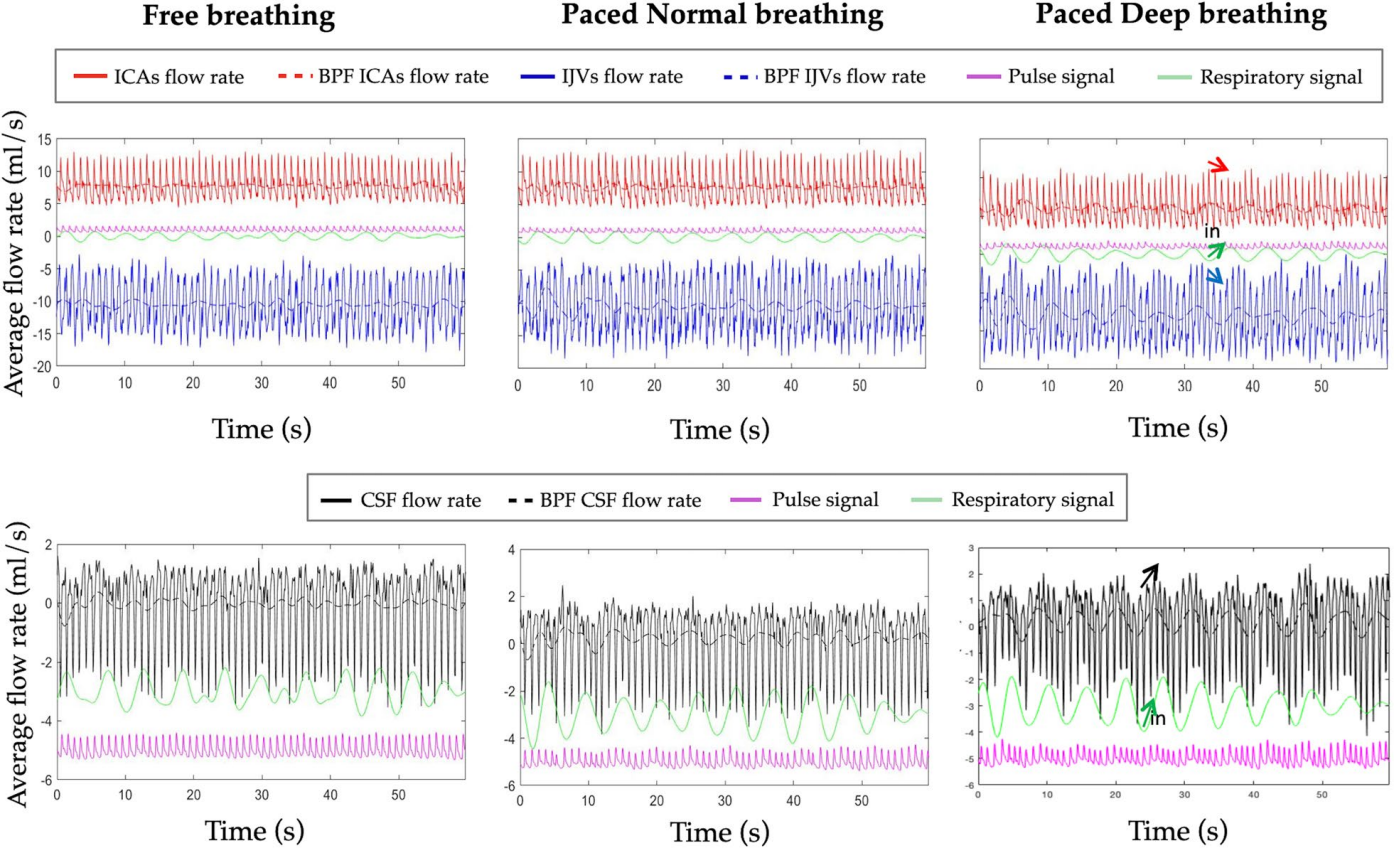
Blood and cerebrospinal fluid (CSF) flow rates aligned with the physiologic signals for the three types of breathing. The flow rate of Internal Carotid Artery (ICA) is represented in red, that of Internal Jugular vein (IJV) in blue and that of cerebrospinal fluid (CSF) in black. The band-pass filtered (BPF) flow rates are superimposed to the respective flow rates for showing their correspondence with respiratory signals. The whole acquisition (60 s) is shown. The effect of inspiration (in, green arrows) on the flow rates are underlined with arrows in the deep breathing, where the respiratory influence is the greatest

Blood and CSF data aligned with the physiologic signals are shown for one exemplificative subject and for the three breathing modes in Fig. 5. The band-pass filtered (0.15–0.35 Hz Hz) flow rates are superimposed to the respective flow rates, for showing their correspondence with respiratory signals.

In Fig. 5 it can be visually see that the mean flow rate was lower in the PD compared to F breathing, as will be statistically shown at the group level hereafter. Taking the breathing mode with the lowest velocity (PD) as exemplificative of low signal, we showed also the ICAs, IJVs, and CSF average velocity in Additional file 1: Figure S1, together with the velocity inside the NFA, that represent the image bias.

A signal sub portion (of about 10 s over 60 s) is also represented in the Additional file 2: Figure S2 to show the signal quality and the morphology of some cardiac beats and to cover a complete respiratory cycle.

Additional file 3: Figure S3 shows a representative pulse wave (separately for ICAs, IJVs, and CSF flow rates): the diastolic peaks were identified over the whole 60-s acquisition and aligned, each heart cycle dynamic was stretched or compressed, normalizing each cardiac cycle duration to the mean HR as previously described in [32], and finally the median, 5th and 95th percentiles of all the curves were computed.

The group averages and respective SD for ICA, IJV and CSF, for F, PN and PD breathing are reported in Table 1. All the paired comparisons between breathing modes were significant (p < 0.001) for the arterial and venous flow rate and their peaks, with the trend F > PN > PD. As regards the CSF, its mean flow rate and absolute flow rate did not significantly change due to mode of breathing, but the CSF systolic peak has the same trend F > PN > PD, and its amplitude is significantly smaller in the PD breathing compared to the other two (Table 1).

**Table 1:** Average flow rate (ml/s) over the 60 s-acquisition and cross-sectional area (mm^2^), for Internal Carotid Artery (ICA), Internal Jugular vein (IJV), and cerebrospinal fluid (CSF)

	Region of interest	F	PN	PD	F vs PN	F vs PD	PN vs PD
Average flow rate (ml/s)	ICA	7.52 ± 1.75	6.06 ± 1.61	4.73 ± 1.25	< 0.001	< 0.001	< 0.001
	IJV	− 7.31 ± 2.29	− 5.74 ± 2.45	− 4.52 ± 2	< 0.001	< 0.001	< 0.001
	CSF	0 ± 0.11	0.02 ± 0.12	0.04 ± 0.11	0	0	0
	Absolute CSF	1.09 ± 0.29	1.12 ± 0.35	1.11 ± 0.39	1	1	1
Cross-sectional area (mm^2^)	ICA	33.23 ± 7.52	33.27 ± 6.15	30.75 ± 5.16	1	0.209	0.038
	IJV	38.21 ± 26.45	37.73 ± 27.67	36.01 ± 24.12	1	0.985	1
	CSF	178.9 ± 53.45	186.31 ± 48.98	182.82 ± 54.34	0.974	1	1
Systolic peak (caudal peak for the CSF)	ICA	10.29 ± 2.44	8.64 ± 2.50	6.73 ± 2.00	< 0.001	< 0.001	< 0.001
	IJV	− 9.08 ± 3.14	− 7.10 ± 3.41	− 5.44 ± 2.65	< 0.001	< 0.001	< 0.001
	CSF	− 1.81 ± 0.60	− 1.83 ± 0.60	1.54 ± 0.57	1	0.016	0.002
Diastolic peak (cranial peak for the CSF)	ICA	5.11 ± 1.34	3.83 ± 1.19	2.79 ± 0.99	< 0.001	< 0.001	0.001
	IJV	− 5.90 ± 2.05	− 4.73 ± 1.76	− 3.91 ± 1.72	< 0.001	< 0.001	0.006
	CSF	1.44 ± 0.53	1.54 ± 0.53	1.49 ± 0.59	0.360	1	1

Mean values ± standard deviation are reported. The free (F), paced normal (PN), paced and deep (PD) respirations paired comparisons are all significant with p < 0.05

The ICAs cross sectional areas were significantly smaller in PD compared to PN breathing (p = 0.038), while the IJVs and CSF cross sectional areas did not significantly change among the various breathing types (Table 1).

### Respiratory and cardiac modulation changes with type of breathing (aim 2 B)

**Fig. 6:**
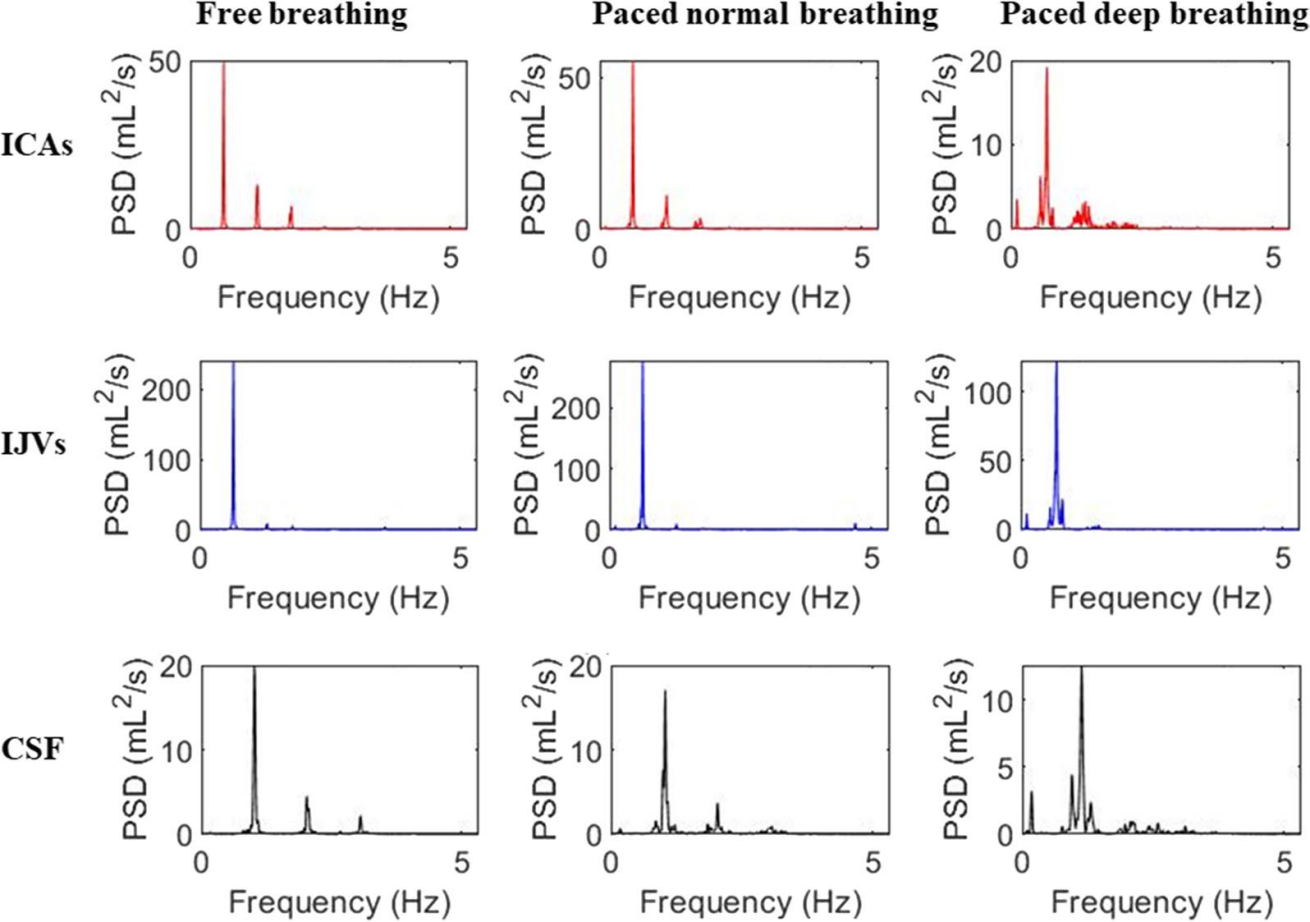
Power spectral density (PSD) of Blood and CSF flow rates during the three types of breathing. PSD are expressed in (ml/s)^2^/Hz = ml^2^/s

The power spectral densities corresponding to the signal shown in Fig. 5 are reported for F, PN and PD breathing in Fig. 6. At visual inspection, an increase of the HF peak and a decrease of the VHF peak with pacing can be observed in spectra shown in Fig. 6. Two spurious peaks at the sides of the fundamental VHF peak, specifically at the frequency of VHF peak ± HF peak, can be also observed in the spectra of PD breathing.

**Fig. 7:**
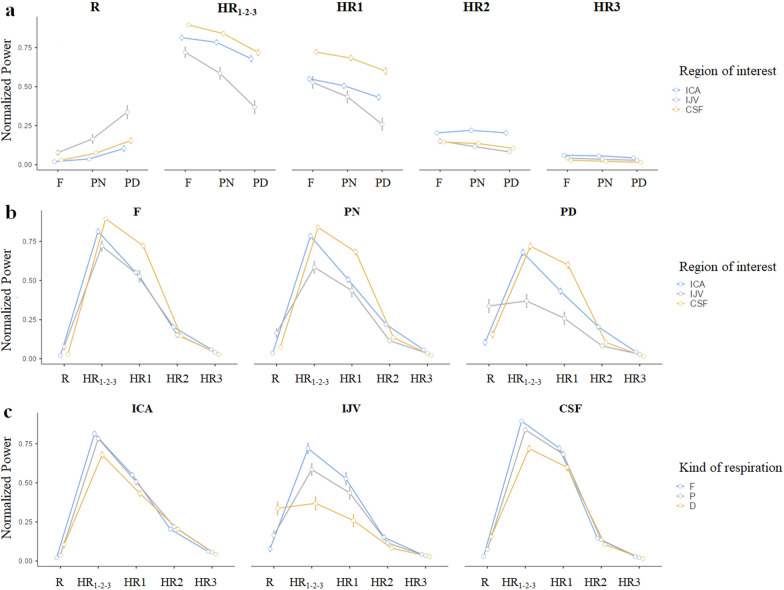
Normalized powers in the HF band (R), VHF band (HR1-2-3) and in the 0.5 Hz-wide bands centered on the 1^st^, 2^nd^, and 3^rd^ VHF peak harmonics (HR1, HR2 and HR3 respectively). The marginal means estimated by the Repeated-Measures Analysis of Variance model (RM-ANOVA) and their standard errors are represented. For a better understanding, the values are grouped for: **a** comparing the kind of respirations (Free—F, Paced Normal—PN and Paced Deep—PD); **b** comparing the normalized powers in the various bands, and the different regions of interest, separately for the various breathing types; **c** comparing the normalized powers in the various bands, and the breathing types, separately for the various regions of interest. The Bonferroni-corrected p-values of the paired comparisons for the R and HR1-2-3 bands are reported in Table 2; the comparisons among R and the HR bands are reported in Additional file 7: Table S4

Some of the normalized powers were not normally distributed. For this reason and for a proper post-hoc analysis, the statistics were performed on the transformed variables, that confirmed to be normally distributed. The statistics on the whole group of subjects showed that the respiratory normalized power significantly increased with pacing, with the greatest values in the PD condition (F < PN < PD) (Table 2 and Fig. 7). The cardiac normalized power, i.e., the sum of the cardiac AUCs under the three cardiac harmonic peaks normalized by the flow rate variance, had an opposite trend, decreasing from F to PN to PD respiration (Table 2 and Fig. 7). This cardiac contribution decrement was mainly guided by the 1st harmonic for all the fluids: it had the same significant trend (F > PN > PD) as the whole cardiac component, with its normalized AUC during PD being always significantly different compared to the one associated with other breathing types (Additional file 7: Table S4 and Fig. 7). The normalized power associate with the 2^nd^ VHF peak harmonic decreased from F to PD breathing for IJVs and CSF only (Additional file 7: Table S4 and Fig. 7).

**Table 2:** Normalized powers in the respiratory (R) and cardiac (HR_1-2–3_) bands, for Internal Carotid Artery (ICA), Internal Jugular vein (IJV), and cerebrospinal fluid (CSF)

	Region of interest	F	PN	PD	F vs PN	F vs PD	PN vs PD
Respiratory band (R)	ICA	0.01 [0.01–0.07]	0.03 [0.01–0.11]	0.07 [0.02–0.44]	< 0.001	< 0.001	< 0.001
	IJV	0.06 [0.01–0.52]	0.10 [0.01–0.49]	0.31 [0.04–0.74]#	< 0.001	< 0.001	< 0.001
	CSF	0.02 [0.01–0.17]	0.05 [0.01–0.28]	0.13 [0.02–0.42]	< 0.001	< 0.001	< 0.001
Cardiac band HR_1-2–3_	ICA	0.84 [0.63–0.92]	0.81 [0.56–0.91]	0.67 [0.35–0.84]	0.489	< 0.001	0.002
	IJV	0.77 [0.25–0.91]	0.63 [0.13–0.88]	0.30 [0.1–0.87]#	< 0.001	< 0.001	< 0.001
	CSF	0.90 [0.78–0.96]	0.87 [0.67–0.93]	0.72 [0.47–0.9]	0.019	< 0.001	< 0.001

Median [range] values are provided. Free (F), paced normal (PN), paced and deep (PD) breathing are compared, and the Bonferroni-corrected p-values of the paired comparisons are reported. The comparisons between the two bands were all significant with p < 0.001, with the exception of #p = 1

Significant differences were observed at paired comparisons of the classic R/C index measured for different breathing conditions for ICA, IJV and CSF (p < 0.001) (Additional file 4: Figure S4). This observation confirmed the respiratory contribution increment with paced and forced breathing, being it the lowest in the F and the greatest in the PD condition.

Comparing the respiratory and the cardiac contributions reported in Table 2, the latter prevailed. Specifically, a significant difference was observed for the majority of paired comparisons, while the only non-significant difference was obtained for IJV during PD breathing (p = 1 for IJV in PD breathing; p = 0.017 for IJVs during the PN condition; p < 0.001 for all the remaining paired comparisons). The respiratory contribution (Additional file 7: Table S4) of IJVs was not significantly lower than the 1st VHF peak during the PN and not significantly different than the 2nd VHF peak during F breathing. The CSF respiratory contribution was significantly greater than the 3rd VHF peak harmonic during the PD. Only for the ICAs, the respiratory contribution was significantly lower even than the 3rd cardiac harmonic.

### Volumes during inspiration vs. expiration (aim 3)

We observed a trend (significant uncorrected statistics, that did not survive the Bonferroni correction for multiple comparisons) of IJVs differential volume increment during inspiration compared to expiration (p = 0.008, uncorrected for multiple comparisons) in PN breathing and in F breathing (p = 0.022), and the same trend in ICAs during the F breathing (p = 0.018) (Additional file 5: Figure S5). No other differential volume was statistically different between the two respiratory phases.

## Discussion

Using a prototype RT-PC [24] sequence with a temporal resolution of 58.5 ms for the blood and 94 ms for the CSF, we were able to estimate the blood and the CSF flow rates while the subjects breathed freely or with a constant rate, with a normal or forced respiration in the latter case. By computing the power spectrum of ICAs, IJVs and CSF flow rates in the three breathing conditions, we could study the respiratory and cardiac influences on the flows.

We previously presented this protocol in [27], but in the current study, acquisition during free breathing was additionally assessed, as it is the normal breathing condition during conventional PC-MRI. We also introduced an analysis of the normalized power in various frequency ranges, representing the respiratory and cardiac contributions to the flow rate variance. Finally, we increased the sample size to 30 healthy subjects.

This setup allowed us to demonstrate that: (1) the HF peak and the VHF peak of the spectrum and their harmonics corresponded to the respiratory and cardiac frequencies and their harmonics; (2) paced, and forced breathing on a greater extent, caused a blood mean flow rate decrement, caudal CSF peak decrement, and an increment of respiratory modulation on all the fluids when compared to free breathing; (3) the differential volumes of ICAs, IJVs, and CSF displaced during inspiration vs. expiration were not statistically different after correcting for multiple comparisons.

The quality of the flow rates vs. time curves are shown for a representative subject in Fig. 5, in the zoomed signals of Additional file 2: Figure S2 and in the representative cardiac cycle of Additional file 3: Figure S3. The quality of the velocity vs. time curves and their relative amplitude compared to the bias velocity are shown in Additional file 1: Figure S1.

The ICAs, IJVs and CSF flow rate changes during inspiration and expiration phases have been qualitatively shown in Fig. 5 on one exemplificative subject: during inspiration (green arrow), the IJVs flow rate increment (blue arrow) and ICAs flow rate decrement (ref arrow), and the CSF caudal flow rate (black arrow) are underlined with arrows.

The increase in IJV flow and decrease in ICA flow during inspiration (Fig. 5) may occur with different phase lag on various subjects, as commented in the paragraph about breathing phase influence on flow volume. It can be explained through pressure forces driven by changes in intrathoracic pressure. Specifically, based on Boyle’s law (P1V1 = P2V2), physical displacement of the diaphragm during inspiration increases the volume of the thoracic/lung cavity and decreases the total thoracic pressure. The resulting decrease in pressure within the right atrium further drives the pressure differential and significantly increases venous preload to the heart (greater jugular flow). On the other hand, the inspiration-induced increase in pulmonary blood volume contributes to lower left ventricular filling resulting in smaller stroke volume and lower ICA flow. The direct relationship between the right and left ventricle acts as an additional pressure/volume loop. Due to the intraventricular dependence, the filling of the right ventricle significantly displaces the left ventricle and prevents larger end-diastolic volume which lowers ICA flow [33, 34].

### Flow rate drivers

The few previous studies that estimated the venous and CSF flow rates using MRI in RT and computed their power spectrum described a HF peak and a VHF peak in the spectra [12, 23, 24]. The frequency of the latter matched to the typical cardiac frequency, while the former corresponded to the respiratory frequency asked to the subject during the experiment. We demonstrated that the HF peak and the VHF peak corresponded to the respiratory and cardiac frequencies measured during the RT-PC MRI acquisition with a thoracic belt and a pulse oximeter, respectively. The cardiac influence on blood and CSF flows is well known: periodic and typical oscillations have been shown and studied using the cardiac-gated PC-MRI that is routinely used [35]. Respiration is the other main driver of blood and CSF flows, due to the pressure changes provided by the thoracic pump [4, 12, 24, 25, 36, 37], as shown by previous studies using RT-PC MRI [12, 24, 25, 37] and ultrasound [4, 36]. The advantage of ultrasound over MRI in these kinds of studies is that it is intrinsically an RT technique. Conversely, the classic PC sequence acquires one segment of k-space lines in one heartbeat, so it requires various cardiac for reconstructing a cardiac cycle: flow can be quantified in RT through MRI with prototypes or customized sequences [24, 38], which mainly restricts to research studies. However, one of the advantages of MRI compared to ultrasound is that it allows the investigation of CSF in addition to blood flow rate. Indeed, CSF respiratory oscillations have been demonstrated using MRI in some previous works [11, 12, 22, 24, 25, 37]. Only a few recent MRI studies showed the respiratory influence on venous flow [11, 12, 39] as well, and its changes with forced breathing. We decided to investigate the respiratory and cardiac influences on CSF, venous and arterial flow rates. To the best of our knowledge, only [11] studied the cardiac and respiratory influences also for the arterial flow, besides the venous and CSF flows. However, in that study an EPI sequence was used, rather than a PC-MRI sequence so the flow could not be quantified. Moreover, in our current study, a temporal resolution that is higher compared to that used in literature was adopted [11, 12, 22, 24, 25, 37].

Given our temporal resolution and image quality, our signals were rich with information, as can be observed in Additional file 2: Figure S2. This allowed us to also verify the presence of the second respiratory harmonic and the 2nd and 3rd cardiac harmonics. As shown for the fundamental harmonics (i.e., HF and VHF peaks), we verified that these other peaks were the respiratory and cardiac harmonics, corresponding to 2*BR, 2*HR, and 3*HR. The differences we found between the 2nd harmonic of the HF peak and the 2*BR for the ICA and CSF in the F condition have to be probably ascribed to low peaks amplitude in F breathing, which may have produced peak misdetection (i.e., a peak of a similar height but of slightly different frequency could have been detected near to the real 2nd harmonic HF peak). The differences detected between the 3rd VHF peak harmonic of the ICA spectrum in the PN and 3*HR could be ascribed to the same reason.

### Breathing type influence on mean flow rates

As recently shown by Kollmeir et al. [12] in 16 healthy subjects, we confirmed the venous flow rate decreases with forced breathing. We also showed that paced breathing compared to free breathing, and forced paced breathing compared to a normal paced respiration, provoked arterial flow rate decrement. The arterial flow rate decrement was concurrent to ICAs cross-sectional area decrement, so it was a consequence of vasoconstriction. Vasoconstriction probably occurred because an increase of oxygen saturation due to respiration strength increment induces a vasocompensatory effect on arteries and reduces arterial flow rate. This physiologic flow autoregulation helps maintain constant brain oxygen. As a consequence of arterial flow rate decrement with paced breathing, we observed that a concurrent decrease in IJVs flow rate, in absence of cross-sectional area decrement.

Conversely to Kollmeir et al. [12], we did not find that average CSF changes with breathing type. Indeed, the average arterial flow decrement in the PD breathing compared to F breathing was well mirrored by venous decrement, but not by a CSF average flow rate change.

Since we expected the blood flow to decrease with forced respiration as in [12] and in our preliminary work [27], and since we wanted to compare the flow rates in three breathing conditions, we designed our study protocol so that each RT-PC acquisition with paced breathing started three breaths after we asked the subject to breathe with our pacing. Our preliminary tests (Additional file 6: Figure S6) revealed that the mean flow rate decreased in a few seconds passing from free to paced breathing, and that after three complete breathing cycles it reached a new regime. This can be slightly observed even when looking at the IJV flow rate over time represented in Fig. 5 of [12]: the venous flow rate progressively decreased from 40 s, when switching from free breathing to forced breathing, and it went lower and then constant from 60 to 80 s (during forced paced breathing). In our current study transitory cycles were excluded from the analysis, so we could compare three “pure” breathing conditions. We acknowledge that our setup (excluding transitory cycles) does not allow to investigate the change rate, while the setup of [12] would potentially allow that. For studying the rate of average flow change in physiological or pathological cases, the subjects should be instructed to breath freely and then with a forced strength during the same sequence.

### Breathing type influence on flow rate modulations

The frequency analysis showed that the respiratory component increased with pace breathing (both PN and PD) when compared to F breathing. Since the flow rate variance was stable during the different breathing types (results not shown), and since it is mainly dependent on the sum of the three cardiac components and the respiratory components, the sum of the powers of the cardiac peaks decreased with paced breathing when compared to free breathing, with an opposite and complementary trend compared to the respiratory power. This means that the flow rate oscillations at the respiratory frequency, i.e., the breathing modulations, become higher, while those at the cardiac frequency become lower in paced breathing with respect to free breathing. The cardiac component remained the prevalent driver in all the types of breathing patterns for ICAs and CSF flow rates, where the normalized cardiac power was consistently higher than the respiratory one. Conversely, cardiac and respiratory components were not statistically different for the IJVs in the PD breathing mode. The powers in the respiratory and cardiac ranges normalized by the signal variance are easy to interpret: they provide respiratory and cardiac flow rate modulations respectively with an index from 0 to 1. Interestingly, from Table it can be observed that respiration accounts up to 50% of the flow rate variance for the IJVs in F and PN breathing and had a maximum of 74% during PD breathing. In the latter breathing mode, the normalized power of respiration had a median value of 31%, similarly to the normalized cardiac power (30%). The respiratory normalized power during the PD breathing had a maximum of more than 40% for ICAs and CSF.

The AUC computed integrating the flow rate over a sufficiently broad bandwidth guaranteed to consider also instable cardiac and respiratory frequencies. So, even in the free breathing, where the frequency can vary across the acquisition, we considered not only a single HF peak but a range around it. For this reason, our results of a higher respiratory modulation in the PD condition compared to the free condition cannot be due to the concentration of the breathing peak on a specific frequency.

Deepening our frequency analysis with a specific evaluation of the three VHF peak harmonics, we observed (Additional file 7: Table S4) that the cardiac contribution decrement was due to the 1st and 2nd harmonic power decrement for IJVs and CSF; conversely it was due to a decrement of only the 1st harmonic for ICAs. Indeed, the normalized power in the first HR harmonic band in the PD breathing was significantly different compared to the other breathing modes for all the structures. Conversely, the normalized power in the second HR harmonic band did not change with breathing mode for ICAs, but was significantly lower in the PD compared to the F breathing for IJVs and CSF. This means that ICAs flow rate changed its shape with PD breathing compared to the other two breathing modes, while the oscillations at the HR and 2*HR both decreased for IJVs and CSF flow rates, not changing their main shape in the PD compared to the F breathing. This analysis also showed that PD respiration increased the respiratory modulation so much that the normalized power of respiration was similar to the normalized power in the 1st (the highest) and to the 2nd (the second highest) cardiac harmonic for IJVs and CSF, respectively. For ICAs, the normalized power of respiration in the PD breathing increased just until the normalized power of the 3rd cardiac harmonic (the smallest one) in the PD respiration. A similar result is shown by the R/C index: it being the ratio between the respiratory and the 1st cardiac powers, it was always below 1 for ICA and CSF, but for some subjects it increased over 1 for IJV with paced breathing (Additional file 4: Figure S4). Similarly, Kollmeir et al. [12] showed R/C below 1 for CSF during normal and forced breathing and for IJVs for normal breathing, but it exceeded 1 for some subjects during forced breathing.

The high respiratory modulation of the venous flow rate during the PD breathing confirmed the results of previous studies [12, 25, 39], since the effect of the thoracic pump increases with forced respiration. Being that venous and CSF flows are highly related [12, 35, 40], the respiratory modulation had a great effect also for the CSF flow rate, and increased with forced breathing when compared to other breathing conditions. We have to underline that with our experiment we demonstrated that the respiration type influences the arterial flow as well.

Two peaks at the edges of the main VHF peak were also observed in paced breathing and were particularly evident for the PD breathing. These are spurious harmonics, due to the interference of the two cyclic flow rates drivers, i.e., respiration and cardiac beats.

### Breathing phase influence on flow volume

As suggested by some previous studies [12, 25], we expected that during inspiration, the CSF flow volume was directed cranially and that the venous flow was greater compared to expiration. However, this hypothesis was confirmed at the uncorrected level only for IJVs during the F and PN breathings and for ICAs during the F breathing (Additional file 5: Figure S5). Specifically, larger volumes were displaced during inspiration compared to expiration (negative differential volume for IJVs and positive differential volumes for ICAs mean higher flow compared to the average). The higher ICA flow volume found in inspiration when the subjects breathed freely counterbalances the larger venous outflow expected during inspiration and guaranteed the mass preservation (Monro-Kellie doctrine, [41]). Similar results were not previously shown using the RT-PC MRI, because the studies in literature investigated venous and CSF flow. As regards the CSF, we did not found different volumes in inspiration vs. expiration. Although such a difference has been qualitatively reported in Kollmeier’s recent paper (Fig. 2) [12], in Akatas [25] (Fig. 3), and in Dreha-Kulaczewski [42] (Fig. 2), associated detailed statistics were not provided. This may justify the discrepancy between our results and those previously reported. In a previous study using ultrasound [4], we reported that blood velocity in some of the examined major neck veins decreased during inspiration and increased during expiration. Although respiration modulated the blood velocity in veins, different kinds of phase synchronizations between flow rate and respiration were observed in real-time with ultrasound. In the current study, we observed the same kind of result in IJVs, but also in CSF and ICAs: we hypothesize that for this reason, the flow volumes were not statistically different after correcting for multiple comparisons, but only some trend could be observed. Specifically, the flow rate was synchronized with respiration only in some subjects, such that the inspiration corresponded to increment in IJVs and ICAs and cranial CSF. For other subjects, a time lag was observed, especially for the CSF, as the flow rate was not synchronously influenced by a particular respiratory phase. This probably prevented us from obtaining significantly different volumes between inspiration and expiration; indeed, only a trend of different volumes between inspiration and expiration was found for IJVs in PN and PD breathing, and for ICAs in PN breathing, for which most of the subjects had the same kind of alignment between respiration and flow rate.

### Strengths and weaknesses

We have confirmed the recent results of Kollmeier et al. [12], in a larger group of subjects: when comparing forced breathing to free breathing, the IJVs flow rate decreases, the respiratory modulation on cervical IJVs and CSF flow rates increases and the cardiac modulation decreases. Differently from the literature about similar measures using MRI, we focused also on the cervical arterial flow, showing that during forced breathing vasoconstriction occurs and that the ICAs flow rate decreases. This may be the probable driver of venous decrease for autoregulation. Investigating ICAs flow rate allowed also to show that in the free breathing, there is a trend for higher ICAs flow volume during inspiration compared to expiration.

However, our study has some limitations. As a general limitation of MRI RT-PC studies, the acquisitions are possible only in the supine position. However, that position deserves investigation, since the venous flow redistributes from IJVs to vertebral veins in the sitting position. Second, we did not measure the CO_2_ partial pressure changes during the various types of breathing, which would have helped the interpretation of the average flow rate change. Third, we separately acquired blood and CSF flows, so we could not investigate their temporal coupling during the various beats and respiratory cycles. However, acquiring both blood and CSF flows with two different VENCs would have decreased the temporal resolution. Therefore, this experimental choice allowed for a higher temporal resolution than the one used in previous studies, for both the blood and CSF flow rates. A balance among temporal resolution, signal-to-noise-ratio, and the necessity to have a good in-plane resolution for segmenting the structures of interest, led to a slice thickness of 8.6 mm.

Another peculiarity of our study design is that we compared different breathing modes: we asked the subjects to breathe not only in free and forced conditions, but also in a paced condition with normal breathing strength. This allowed us to confirm that forced breathing increases the respiratory modulations to flow rates, but also to evidence that the respiratory contribution can be enhanced with paced breathing.

We reported how breathing affects the arterial, venous and CSF average flow rates and their modulations in normal volunteers. Differently from previous studies, we described the power spectra normal values detailing different cardiac contributions: from the 1^st^ to the 3^rd^ HR harmonics. We computed the respiratory power normalized by the power in the 1^st^ HR harmonic to compare our results with the same index used in literature, however we also introduced the normalized power in the various frequency range. This index between 0 and 1 represents the contribution of oscillations at a particular frequency range.

Considering that altered blood flow, and CSF net volume or peak velocity were described in many pathological conditions [21, 43, 44], the application of the current protocol into clinical studies might allow to investigate if there are differences in terms of how the flows in/out of the brain are modulated by cardiac beating and by breathing patterns compared to normal values. The current setup could also allow to study if breathing manoeuvre exacerbate CSF flow alterations, and if respiratory exercises allow to normalize blood and CSF flows. The clinical studies might consider to include also other CSF volumetric measures proposed in literature (forward, backward, net and stroke volumes stroke and net volumes) [31, 45], paying attention when interpreting their results. Indeed, the previously cited volumetric measures are computed in one cardiac cycle obtained from the conventional PC-MRI, that requires several heartbeats to reconstruct one cardiac cycle, not considering potential changes due to respiration.

Indeed, forced breathing at a constant rate was proposed [46] as a clinical non-invasive manoeuvre for testing cerebral autoregulation, since it produced sinusoidal blood pressure oscillations at the respiratory frequency, then transmitted to the cerebral blood flow volume with different lags depending on the autoregulation response. There are further numerous possible clinical applications of this study, such as investigating the impact of respiration type for drug delivery through CSF [47, 48]. The continuous blood flow measures through RT-PC MRI could be an alternative way to measure the cerebral blood flow instead of transcranial Doppler, for monitoring the Monro-Kellie Doctrine in RT, as proposed in [49, 50] using ultrasound. Modifying the RT-PC sequences for the contemporaneous acquisition of the blood and CSF flow rate, would even strengthen the RT volume balance computation.

Finally, this set-up could be used to study if blood and CSF circulations are altered in pathological respiration patterns, such as in patients with chronic obstructive pulmonary disease[51], or in obstructive sleep apnea [52].

## Conclusions

RT-PC MRI allowed us to measure the pulsatile arterial and venous blood flow and the oscillatory CSF flow at different breathing modes, and that their relative amplitude depended on the kind of breathing. We also confirmed in a group of 30 healthy volunteers that the blood flow decreased with forced breathing when compared to free breathing. The spectral analyses demonstrated higher respiratory modulations in the PD breathing than in F and PN breathing, due to the greater thoracic pump effect on the flow rates.

We found different kinds of lags between inspiration and flow increment in the IJVs, and between inspiration and cranial CSF flows. The reasons for the intersubject variability of the phase between respiration and flow rate warrant further investigation.

## Supplementary Information

**Additional file 1: Figure S1.** Signal and noise. Signals: average velocities of Internal Carotid Arteries (ICAs) (red), Internal Jugular Veins (IJVs) (blue), and Cerebrospinal Fluid (CSF) (black). Noise: velocity inside the No-Flow-Area (NFA) (maroon) is superimposed to signals to appreciate their different amplitudes, and separately shown to better display their course

**Additional file 2: Figure S2**. Internal Carotid Arteries (ICAs), Internal Jugular Veins (IJVs) and cerebrospinal fluid (CSF) flow rates sub portion (about 10 s), covering two whole respiratory cycles, to better show the details of some cardiac cycles. Free breathing and paced deep breathing are shown

**Additional file 3: Figure S3.** Pulse waves of Internal Carotid Arteries (ICAs), Internal Jugular Veins (IJVs) and cerebrospinal fluid (CSF) flow rates from diastolic to systolic peaks: median, 5th and 95th percentiles of all the curves measured over the 60 s

**Additional file 4: Figure S4.** Power in the low-frequency band (R), normalized for that in the first high-frequency band (HR1) for Internal Carotid Artery (ICA), Internal Jugular vein (IJV), and cerebrospinal fluid (CSF). The marginal means estimated by the Repeated-Measures Analysis of Variance model (RM-ANOVA) and their standard errors are represented. The kind of respirations (Free—F, Paced Normal—PN and Paced Deep—PD) are compared. All the comparisons among kinds of breathing are significant (p < 0.001)

**Additional file 5: Figure S5.** Differential volumes (in ml) during inspiration (in) and expiration (ex), for the Internal Carotid Arteries (ICAs), Internal Jugular vein (IJVs) and cerebrospinal fluid (CSF), separately for the free (F), paced normal (PN), and paced deep (PD) breathing pattern. The p-values reported in the Figure refer to uncorrected statistics. All the comparisons were not statistically significant after correcting for multiple comparisons

**Additional file 6: Figure S6.** Internal Carotid Arteries (ICAs) and Internal Jugular vein (IJV) flow rate: transition from free to paced deep respiration

**Additional file 7: Table S1.** Respiratory and cardiac frequencies (in Hz) during the blood and CSF acquisitions with free (F), paced normal (PN), and paced deep (PD) breathing. The frequencies were obtained from the physiologic signals (thoracic belt and pulse oximeter) and compared among the different modes of breathing. Mean values ± standard deviation are shown. Post-hoc Bonferroni-corrected p-values are reported. **Table S2.** Comparisons between the frequencies measured with the thoracic belt (and its multiples) and the 1st and 2nd HF peak harmonics. Mean values ± standard deviation are reported. **Table S3.** Comparisons between frequencies (in Hz) measured with the pulse oximeter (and its multiples) and the 1^st^, 2^nd^ and 3^rd^ VHF peak harmonics of the power spectral density of the Internal Carotid Artery (ICA), Internal Jugular vein (IJV), cerebrospinal fluid (CSF). Mean values ± standard deviation are separately reported for the free (F), paced normal (PN), paced and deep (PD) respirations. All the comparisons are not significant, with the exception of *p = 0.009. **Table S4.** Normalized powers in the HF band (R), and in the 0.5 Hz-wide bands centered on the 1^st^, 2^nd^, and 3^rd^ VHF peak harmonics (HR1, HR2 and HR3 respectively), for Internal Carotid Artery (ICA), Internal Jugular vein (IJV), and cerebrospinal fluid (CSF). Median[range] values are provided. Free (F), paced normal (PN), paced and deep (PD) respirations are compared, and the Bonferroni-corrected p-values of the paired comparisons are reported. Normalized powers were compared also among various bands: we underlined the pairs of comparisons that were not significantly different with the letters from a to i (a,c,d,e,f,g,h,i:p = 1; b: p = 0.324), since they are useful to understand the trend of the power in the R band compared to the cardiac sub-harmonic; all the other pairs are significantly different

## Data Availability

The datasets analysed during the current study are available from the corresponding author on reasonable request.
