# Supplementary figures and images for: Blood and cerebrospinal fluid flow oscillations measured with real-time phase-contrast MRI: breathing mode matters
Source: Fluids Barriers CNS. 2022 Dec 14;19:100. doi: 10.1186/s12987-022-00394-0 (PMC9749305; doi:10.1186/s12987-022-00394-0)

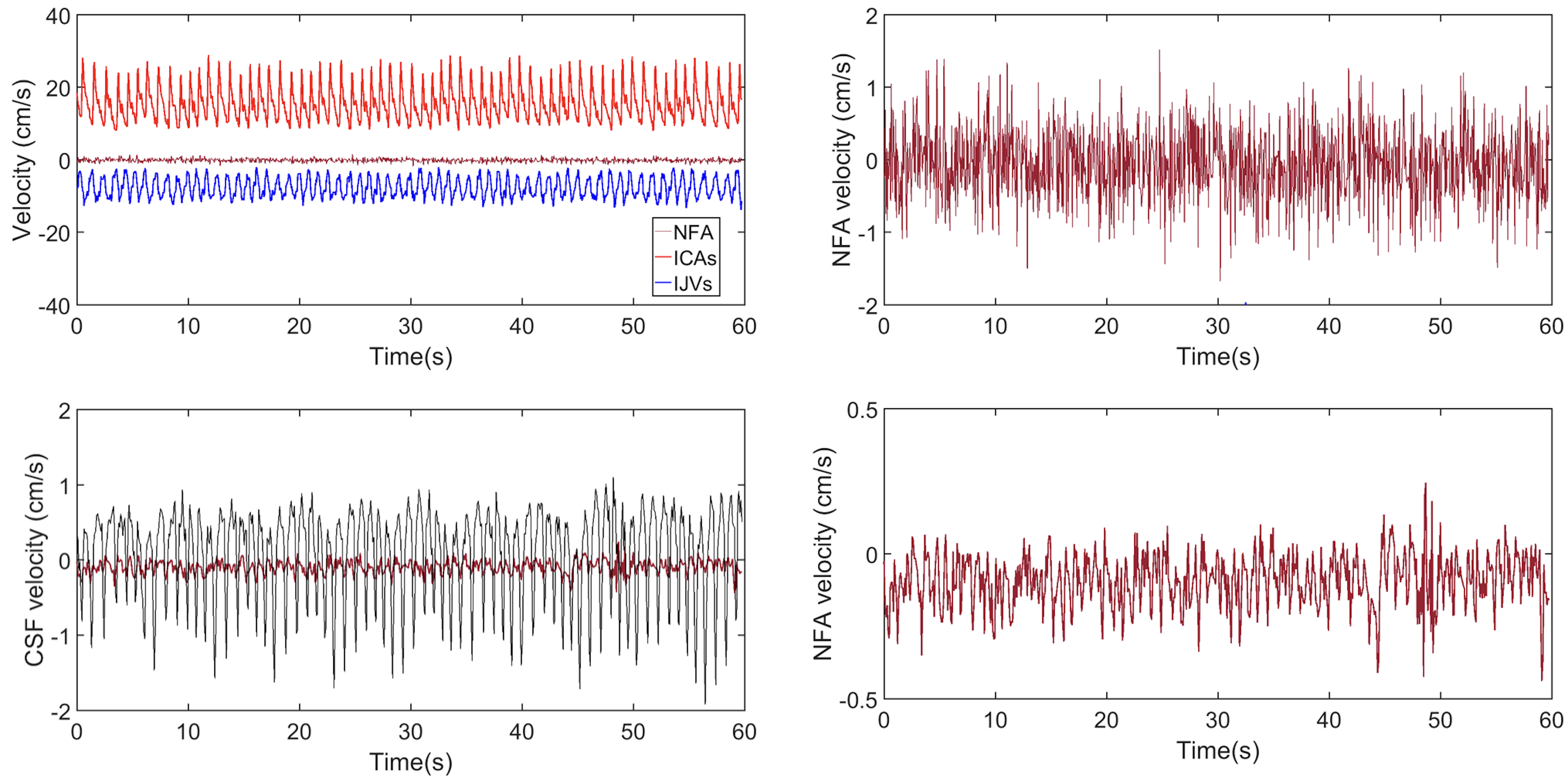

Supplement: Supplementary file 1 — Additional file 1: Figure S1. Signal and noise. Signals: average velocities of Internal Carotid Arteries (ICAs) (red), Internal Jugular Veins (IJVs) (blue), and Cerebrospinal Fluid (CSF) (black). Noise: velocity inside the No-Flow-Area (NFA) (maroon) is superimposed to signals to appreciate their different amplitudes, and separately shown to better display their course [file 12987_2022_394_MOESM1_ESM.png]

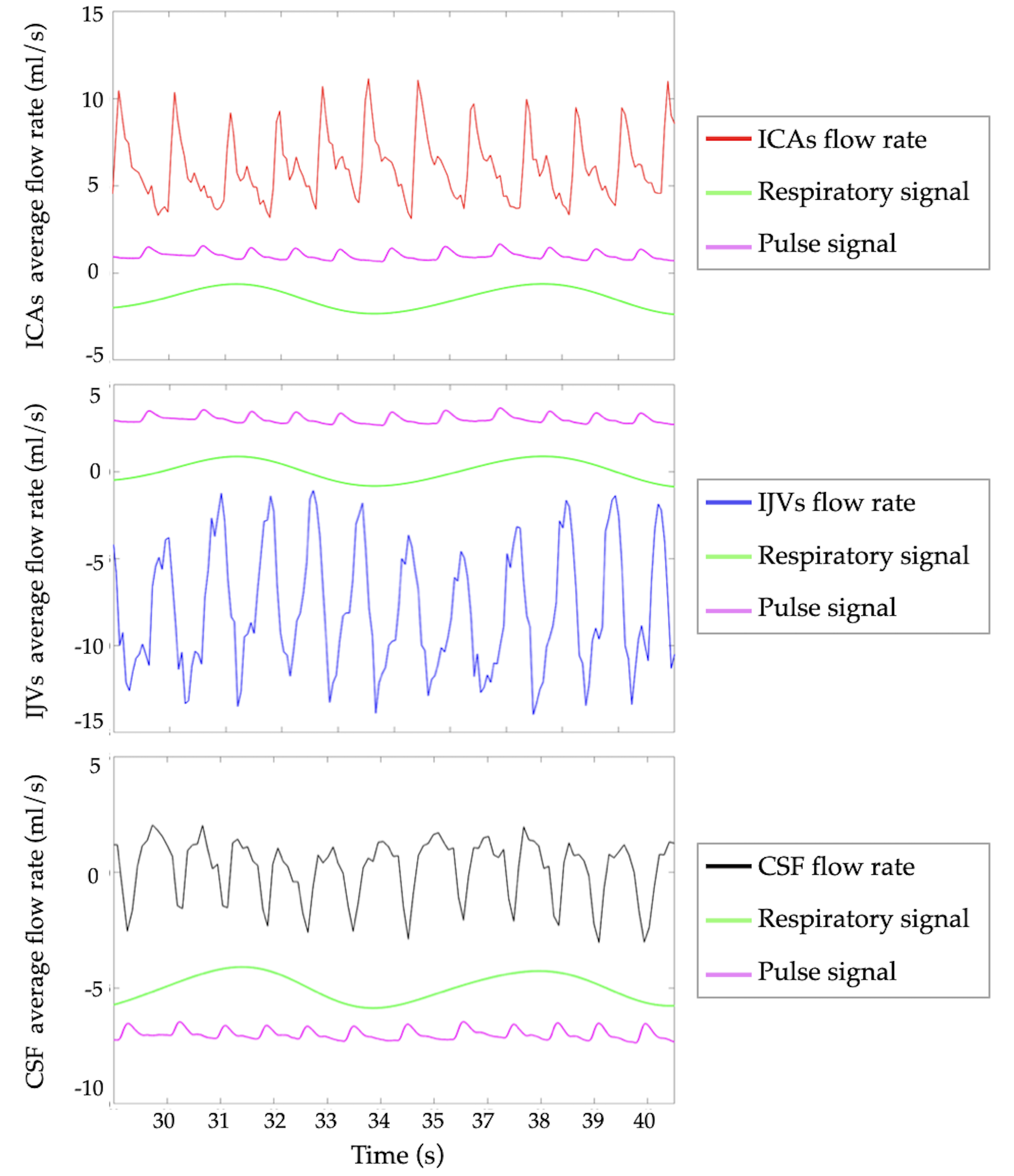

Supplement: Supplementary file 2 — Additional file 2: Figure S2. Internal Carotid Arteries (ICAs), Internal Jugular Veins (IJVs) and cerebrospinal fluid (CSF) flow rates sub portion (about 10 s), covering two whole respiratory cycles, to better show the details of some cardiac cycles. Free breathing and paced deep breathing are shown [file 12987_2022_394_MOESM2_ESM.png]

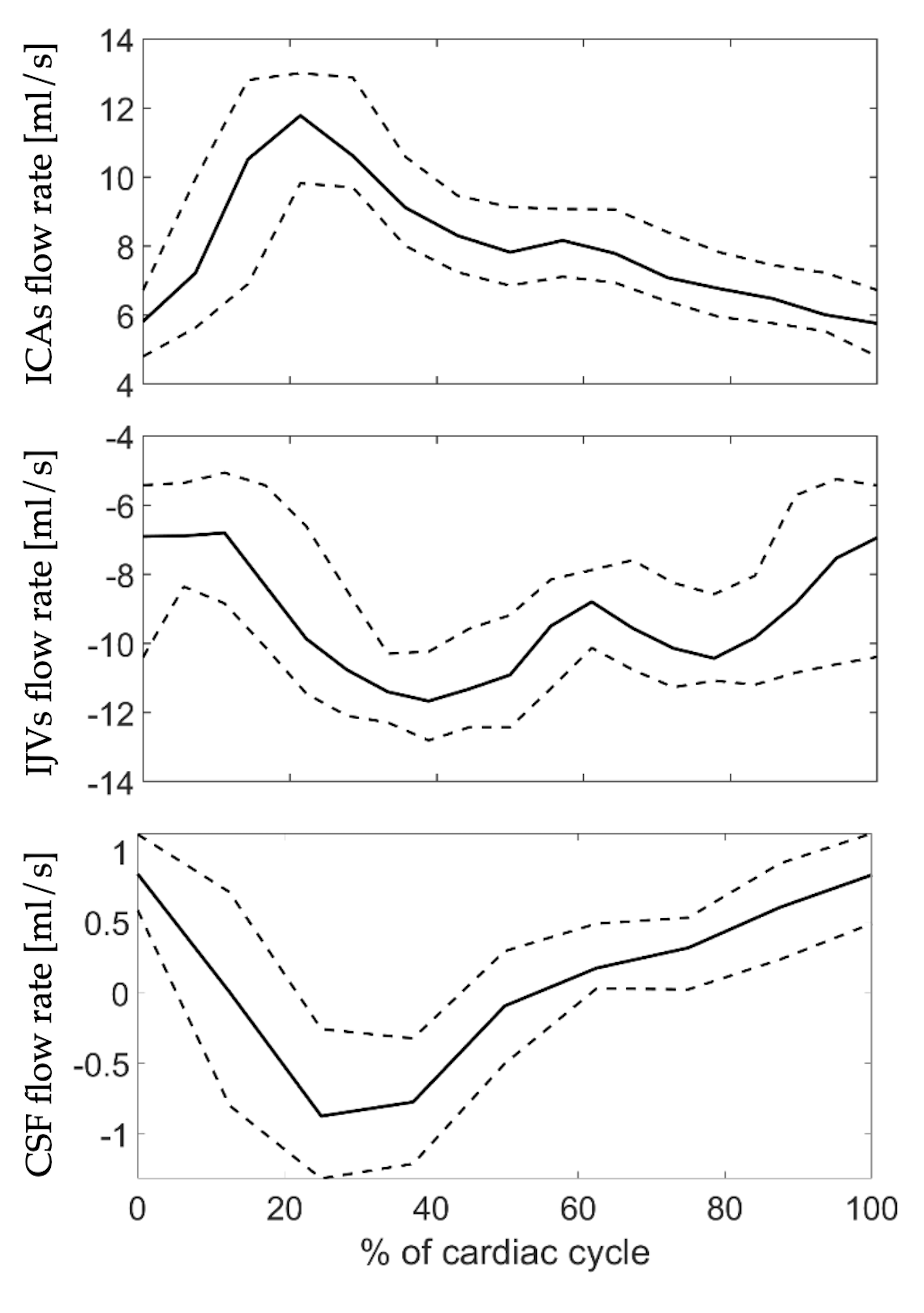

Supplement: Supplementary file 3 — Additional file 3: Figure S3. Pulse waves of Internal Carotid Arteries (ICAs), Internal Jugular Veins (IJVs) and cerebrospinal fluid (CSF) flow rates from diastolic to systolic peaks: median, 5th and 95th percentiles of all the curves measured over the 60 s [file 12987_2022_394_MOESM3_ESM.png]

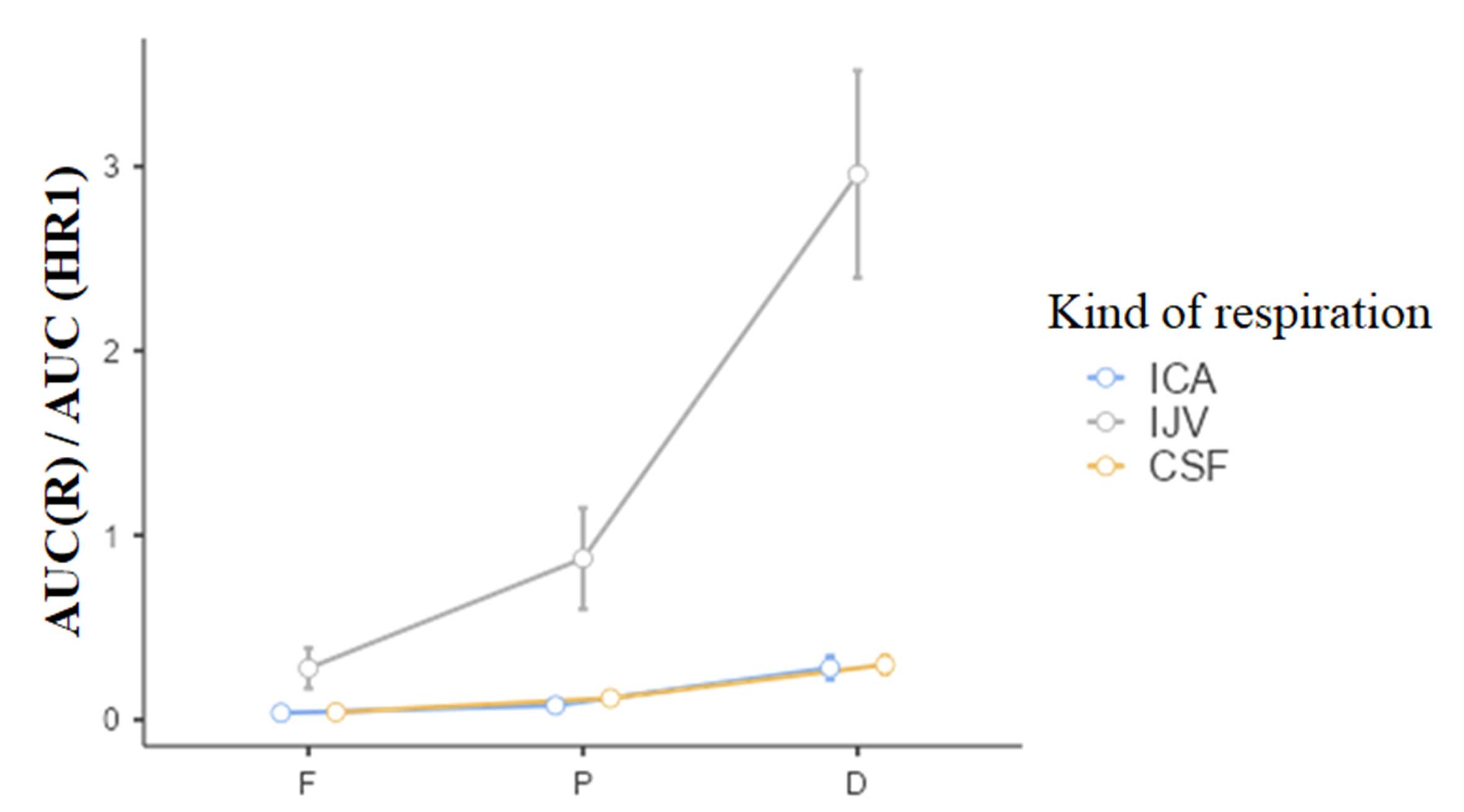

Supplement: Supplementary file 4 — Additional file 4: Figure S4. Power in the low-frequency band (R), normalized for that in the first high-frequency band (HR1) for Internal Carotid Artery (ICA), Internal Jugular vein (IJV), and cerebrospinal fluid (CSF). The marginal means estimated by the Repeated-Measures Analysis of Variance model (RM-ANOVA) and their standard errors are represented. The kind of respirations (Free—F, Paced Normal—PN and Paced Deep—PD) are compared. All the comparisons among kinds of breathing are significant (p < 0.001) [file 12987_2022_394_MOESM4_ESM.png]

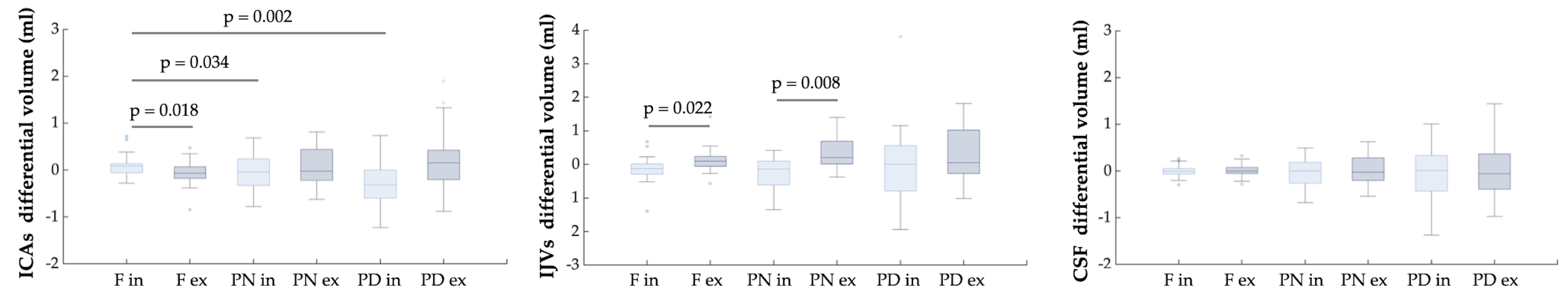

Supplement: Supplementary file 5 — Additional file 5: Figure S5. Differential volumes (in ml) during inspiration (in) and expiration (ex), for the Internal Carotid Arteries (ICAs), Internal Jugular vein (IJVs) and cerebrospinal fluid (CSF), separately for the free (F), paced normal (PN), and paced deep (PD) breathing pattern. The p-values reported in the Figure refer to uncorrected statistics. All the comparisons were not statistically significant after correcting for multiple comparisons [file 12987_2022_394_MOESM5_ESM.png]

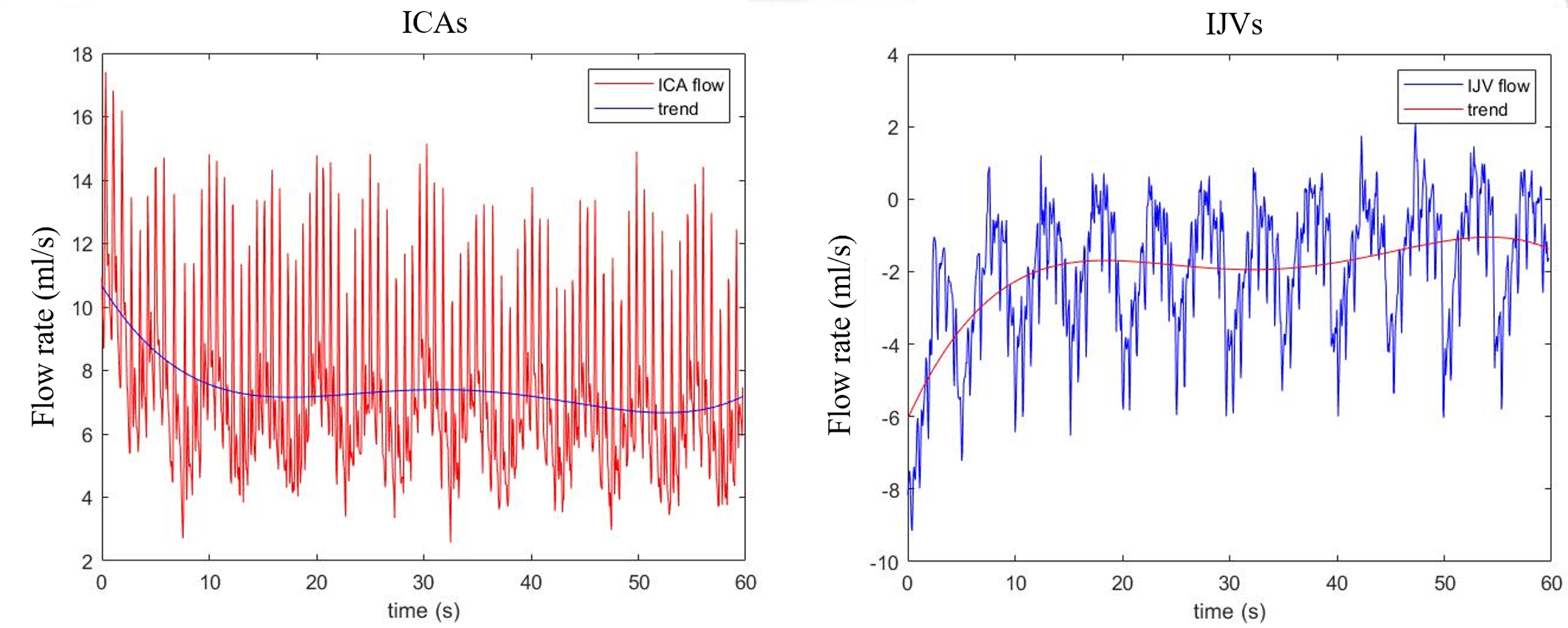

Supplement: Supplementary file 6 — Additional file 6: Figure S6. Internal Carotid Arteries (ICAs) and Internal Jugular vein (IJV) flow rate: transition from free to paced deep respiration [file 12987_2022_394_MOESM6_ESM.png]
