# Supplementary material for: Blood and cerebrospinal fluid flow oscillations measured with real-time phase-contrast MRI: breathing mode matters
Source: Fluids Barriers CNS. 2022 Dec 14;19:100. doi: 10.1186/s12987-022-00394-0 (PMC9749305; doi:10.1186/s12987-022-00394-0)
Supplement: Supplementary file 7 — Additional file 7: Table S1. Respiratory and cardiac frequencies (in Hz) during the blood and CSF acquisitions with free (F), paced normal (PN), and paced deep (PD) breathing. The frequencies were obtained from the physiologic signals (thoracic belt and pulse oximeter) and compared among the different modes of breathing. Mean values ± standard deviation are shown. Post-hoc Bonferroni-corrected p-values are reported. Table S2. Comparisons between the frequencies measured with the thoracic belt (and its multiples) and the 1st and 2nd HF peak harmonics. Mean values ± standard deviation are reported. Table S3. Comparisons between frequencies (in Hz) measured with the pulse oximeter (and its multiples) and the 1st, 2nd and 3rd VHF peak harmonics of the power spectral density of the Internal Carotid Artery (ICA), Internal Jugular vein (IJV), cerebrospinal fluid (CSF). Mean values ± standard deviation are separately reported for the free (F), paced normal (PN), paced and deep (PD) respirations. All the comparisons are not significant, with the exception of *p = 0.009. Table S4. Normalized powers in the HF band (R), and in the 0.5 Hz-wide bands centered on the 1st, 2nd, and 3rd VHF peak harmonics (HR1, HR2 and HR3 respectively), for Internal Carotid Artery (ICA), Internal Jugular vein (IJV), and cerebrospinal fluid (CSF). Median[range] values are provided. Free (F), paced normal (PN), paced and deep (PD) respirations are compared, and the Bonferroni-corrected p-values of the paired comparisons are reported. Normalized powers were compared also among various bands: we underlined the pairs of comparisons that were not significantly different with the letters from a to i (a,c,d,e,f,g,h,i:p = 1; b: p = 0.324), since they are useful to understand the trend of the power in the R band compared to the cardiac sub-harmonic; all the other pairs are significantly different [file 12987_2022_394_MOESM7_ESM.docx]

**Additional file Table 1.** Respiratory and cardiac frequencies (in Hz) during the blood and CSF acquisitions with free (F), paced normal (PN), and paced deep (PD) breathing. The frequencies were obtained from the physiologic signals (thoracic belt and pulse oximeter) and compared among the different modes of breathing. Mean values±standard deviation are shown. Post-hoc Bonferroni-corrected p-values are reported.

|  |  | **Types of respiration** | | |  | **p-values** | | |
| --- | --- | --- | --- | --- | --- | --- | --- | --- |
| **Frequency** | **Acquisition** | **F** | **PN** | **PD** |  | **F *vs* PN** | **F *vs* PD** | **PN *vs* PD** |
| Respiratory | Blood | 0.27±0.03 | 0.24±0.03 | 0.22±0.02 |  | <0.001 | <0.001 | 0.002 |
|  | CSF | 0.27±0.04 | 0.24±0.03 | 0.22±0.03 |  | 0.005 | <0.001 | 0.008 |
| Cardiac | Blood | 1.22±0.22 | 1.28±0.22 | 1.32±0.25 |  | 1 | 1 | 0.314 |
|  | CSF | 1.23±0.24 | 1.32±0.22 | 1.37±0.23 |  | 0.426 | 0.068 | 1 |

**Additional file Table 2.** Comparisons between the frequencies measured with the thoracic belt (and its multiples) and the 1^st^ and 2^nd^ HF peak harmonics. Mean values±standard deviation are reported.

|  | Method | F | PN | PD |
| --- | --- | --- | --- | --- |
|  |  |  |  |  |
| Blood acquisition | Thoracic belt | 0.27±0.03* | 0.27±0.03 | 0.27±0.03 |
|  | ICA 1st HF peak harmonic | 0.26±0.05 | 0.24±0.03 | 0.21±0.03 |
|  | IJV 1st HF peak harmonic | 0.27±0.05 | 0.24±0.04 | 0.22±0.04 |
|  | ICA 2nd HF peak harmonic | 0.5±0.09* | 0.48±0.07 | 0.42±0.07 |
|  | IJV 2nd HF peak harmonic | 0.53±0.1 | 0.49±0.06 | 0.44±0.06 |
|  |  |  |  |  |
| CSF acquisition | Thoracic belt | 0.27±0.04# | 0.24±0.03 | 0.22±0.03 |
|  | CSF 1st HF peak harmonic | 0.26±0.05 | 0.24±0.03 | 0.21±0.03 |
|  | CSF 2nd HF peak harmonic | 0.5±0.09# | 0.48±0.07 | 0.42±0.07 |

**Additional file Table 3.** Comparisons between frequencies (in Hz) measured with the pulse oximeter (and its multiples) and the 1^st^, 2^nd^ and 3^rd^ VHF peak harmonics of the power spectral density of the Internal Carotid Artery (ICA), Internal Jugular vein (IJV), cerebrospinal fluid (CSF). Mean values ± standard deviation are separately reported for the free (F), paced normal (PN), paced and deep (PD) respirations. All the comparisons are not significant, with the exception of *p=0.009.

|  | Method | F | PN | PD |
| --- | --- | --- | --- | --- |
|  |  |  |  |  |
| Blood acquisition | Pulse oximeter | 1.22±0.22 | 1.28±0.22* | 1.32±0.25 |
|  | ICA 1st VHF peak harmonic | 1.2±0.2 | 1.26±0.2 | 1.31±0.23 |
|  | IJV 1st VHF peak harmonic | 1.30±0.45 | 1.30±0.45 | 1.29±0.21 |
|  | ICA 2nd VHF peak harmonic | 2.42±0.41 | 2.54±0.41 | 2.6±0.49 |
|  | IJV 2nd VHF peak harmonic | 2.38±0.42 | 2.46±0.44 | 2.66±0.43 |
|  | ICA 3rd VHF peak harmonic | 3.63±0.62 | 3.72±0.69* | 3.9±0.72 |
|  | IJV 3rd VHF peak harmonic | 3.53±0.7 | 3.69±0.73 | 3.83±0.87 |
|  |  |  |  |  |
| CSF acquisition | Pulse oximeter | 1.23±0.24 | 1.32±0.22 | 1.37±0.23 |
|  | CSF 1st VHF peak harmonic | 1.24±0.23 | 1.27±0.2 | 1.37±0.22 |
|  | CSF 2nd VHF peak harmonic | 2.5±0.43 | 2.55±0.42 | 2.72±0.43 |
|  | CSF 3rd VHF peak harmonic | 3.56±0.7 | 3.75±0.59 | 3.98±0.79 |

**Additional file Table 4.**  Normalized powers in the HF band (R), and in the 0.5Hz-wide bands centered on the 1^st^, 2^nd^, and 3^rd^ VHF peak harmonics (HR1, HR2 and HR3 respectively), for Internal Carotid Artery (ICA), Internal Jugular vein (IJV), and cerebrospinal fluid (CSF). Median[range] values are provided. Free (F), paced normal (PN), paced and deep (PD) respirations are compared, and the Bonferroni-corrected p-values of the paired comparisons are reported. Normalized powers were compared also among various bands: we underlined the pairs of comparisons that were not significantly different with the letters from a to i (a,c,d,e,f,g,h,i:p=1; b: p=0.324), since they are useful to understand the trend of the power in the R band compared to the cardiac sub-harmonic; all the other pairs are significantly different.

| Band | Region of interest | F | PN | PD | F vs PN | F vs PD | PN vs PD |
| --- | --- | --- | --- | --- | --- | --- | --- |
| R | ICA | 0.01[0.01-0.07] | 0.03[0.01-0.11] | 0.07[0.02-0.44]a | **0.005** | **<0.001** | **<0.001** |
|  | IJV | 0.06[0.01-0.52]b,c | 0.1[0.01-0.49]d,e | 0.31[0.04-0.74]f,g | **0.002** | **<0.001** | 1 |
|  | CSF | 0.02[0.01-0.17]h | 0.05[0.01-0.28] | 0.13[0.02-0.42]i | **0.014** | **<0.001** | **0.004** |
| HR1 | ICA | 0.56[0.42-0.67] | 0.53[0.29-0.66] | 0.44[0.25-0.62] | 0.090 | **<0.001** | **0.001** |
|  | IJV | 0.57[0.13-0.83] | 0.45[0.07-0.79]d | 0.16[0.05-0.81]f | 0.398 | **<0.001** | **0.004** |
|  | CSF | 0.73[0.53-0.87] | 0.69[0.47-0.84] | 0.61[0.37-0.77] | 0.858 | **0.001** | **0.033** |
| HR2 | ICA | 0.2[0.1-0.44] | 0.22[0.1-0.43] | 0.21[0.07-0.38] | 1 | 1 | 1 |
|  | IJV | 0.14[0.03-0.36]b | 0.11[0.02-0.26]e | 0.06[0.01-0.24]g | 1 | **0.003** | 1 |
|  | CSF | 0.14[0.05-0.27] | 0.13[0.05-0.27] | 0.09[0.05-0.2]i | 1 | **0.039** | 1 |
| HR3 | ICA | 0.06[0.01-0.15] | 0.05[0.01-0.17] | 0.04[0.01-0.17]a | 1 | 0.8 | 0.247 |
|  | IJV | 0.03[0.01-0.13]c | 0.03[0.01-0.08] | 0.02[0-0.1] | 1 | 1 | 1 |
|  | CSF | 0.02[0-0.14]h | 0.01[0-0.1] | 0.01[0-0.11] | 1 | 0.440 | 1 |
